# Acute and Intermittent Exogenous Ketosis to Support Recovery From Exercise and Adaptations to Exercise Training: A Narrative Review

**DOI:** 10.1111/sms.70158

**Published:** 2025-11-13

**Authors:** Brendan Egan

**Affiliations:** ^1^ School of Health and Human Performance Dublin City University Dublin Ireland; ^2^ Florida Institute for Human and Machine Cognition Pensacola Florida USA

**Keywords:** angiogenesis, erythropoietin, inflammation, muscle glycogen, muscle protein synthesis, rehydration, sleep

## Abstract

The ketone bodies acetoacetate (AcAc) and β‐hydroxybutyrate (βHB) have pleiotropic effects in multiple organs including the brain, heart, and skeletal muscle by serving as an alternative substrate for energy provision, and by acting as a signaling molecule modulating inflammation, oxidative stress, anabolic and catabolic processes, and gene expression. Ketogenic precursors such as 1,3‐butanediol and medium‐chain fatty acids, and ketone body‐containing compounds such as ketone salts and ketone esters are commercially available and collectively termed exogenous ketone supplements (EKS). Ingestion of EKS produces an acute transient (1–2 h) increase in circulating AcAc and βHB concentrations, which has been termed “acute nutritional ketosis” or “intermittent exogenous ketosis”. Many studies have failed to observe benefits of acute ingestion of EKS on various tests of exercise capacity and performance, but recent studies, albeit small in number, have suggested beneficial effects of EKS on recovery, sleep, overreaching, and adaptation to exercise training. This review describes the rationale and potential mechanistic basis for the proposed effects of EKS on these outcomes, as well as critically appraising the existing literature in this field, which at present is largely exploratory and requires more human studies with ecologically valid designs to address important knowledge gaps.

## 1 Introduction

The ketone bodies (KBs) acetoacetate (AcAc) and β‐hydroxybutyrate (βHB) are lipid‐derived, water‐soluble organic compounds produced almost exclusively in the liver, and whose production is amplified most obviously during physiological states characterized by low carbohydrate (CHO) availability i.e., prolonged fasting, starvation, or adherence to ketogenic diets [1, 2, 3]. In vivo administration of KBs from exogenous sources for parenteral nutrition [4], and broad therapeutic applications [5] has been of long‐standing interest. In the past decade, the emergence of commercially available ingestible forms for in vivo administration, collectively termed exogenous ketone supplements (EKS), has catalyzed research on the effects of these supplements in athletic contexts [6, 7]. Included under this broad classification are ketogenic precursors such as 1,3‐butanediol (BD) and medium‐chain fatty acids (MCFA) and triglycerides (MCT), isolated KBs in the form of R,S‐βHB and R‐βHB salts, and ketone esters (Table 1). Ketone esters, which typically consist of a KB esterified to a ketogenic precursor such as a BD, have been prominent in the exercise science literature [7], with the most widely studied form being the (R)‐3‐hydroxybutyl (R)‐3‐hydroxybutyrate (R‐BD R‐βHB) ketone monoester (KME) [7].

**TABLE 1:** Types of exogenous ketone supplements.

Supplement type	Brief overview
Medium chain fatty acids (MCFA)/Medium chain triglycerides (MCT)	MCTs contain FAs that are 6–12 carbons in length (i.e., MCFAs), and examples include caproic acid (C6), caprylic acid (C8), capric acid (C10), and lauric acid (C12) MCFAs can be absorbed via hepatic portal circulation and enter the hepatic mitochondria without requiring carnitine transport, where they are rapidly metabolized to acetyl CoA, and subsequently to KBs
1,3‐butanediol (BD)	BD is converted to β‐hydroxybutyraldehyde in the liver, and oxidized to produce βHB via the actions of alcohol dehydrogenase and aldehyde dehydrogenase, respectively Ingestion of BD can increase circulating [βHB] in isolation, or can augment increases in circulating [βHB] in response to ketone ester ingestion when present as an esterified component of the compound
Ketone salts (KS)	KS are typically a racemic mixture of R,S‐βHB, but can also be non‐racemic βHB salts or enantiopure R‐βHB molecules The KB is bound to a mineral salt or a combination of mineral salts, such as calcium, sodium, or potassium
(R)‐3‐hydroxybutyl (R)‐3‐hydroxybutyrate (R‐BD R‐βHB) ketone monoester (KME)	A ketone monoester produced by synthesis of R‐β‐hydroxybutyrate and R‐1,3‐butanediol This ketone ester is salt‐free, has 99% chiral purity, and therefore only provides the R form of βHB
R,S‐1,3‐butanediol acetoacetate (R,S‐BD AcAc) ketone diester (KDE)^a^	A ketone diester produced by transesterification of t‐butylacetoacetate with R,S‐1,3‐butanediol This ketone ester is a non‐ionized sodium‐free and pH‐neutral precursor of AcAc
Bis hexanoyl (R)‐1,3‐butanediol (BH‐BD) ketone diester (KDE)^a^	A ketone diester of hexanoic acid (a 6‐carbon ketogenic MCFA also known as caproic acid) and R‐1,3‐butanediol
Bis‐octanoyl (R)‐1,3‐butanediol (BO‐BD) ketone diester (KDE)	A ketone diester of octanoic acid (an 8‐carbon ketogenic MCFA also known as caprylic acid) and R‐1,3‐butanediol

Abbreviations: βHB, β‐hydroxybutyrate; AcAc, acetoacetate; FA, fatty acid; KB, ketone body.

^a^ Not currently (Q3 2025) commercially available.

Ingestion of EKS in athletic contexts is undertaken primarily with the aim of elevating circulating [R‐βHB] to achieve a state termed “acute nutritional ketosis” [8] or “intermittent exogenous ketosis” [9, 10]. This effect can occur within minutes of ingestion and be maintained for several hours depending on the type and dose of EKS [7]. Acute or intermittent exogenous ketosis has been consistently observed to have obvious effects on metabolism at rest, and during and after exercise [3, 6, 7, 11]. These effects have led to considerable interest in EKS as beneficial compounds in the contexts of athletic performance, recovery and beyond [6, 12, 13, 14, 15, 16, 17, 18, 19, 20, 21].

While a handful of studies have shown benefits of acute ingestion of EKS on athletic performance [22, 23, 24, 25], two of which have required co‐ingestion with sodium bicarbonate [23, 25], the majority of studies have failed to observe a benefit [9, 26, 27, 28, 29, 30, 31, 32, 33, 34, 35, 36, 37, 38]. Negative effects on parameters of exercise performance and maximal exercise testing have also been observed in several studies [39, 40, 41, 42, 43, 44, 45]. Given that the overall evidence base at present demonstrates little benefit for acute ingestion of EKS on tests of exercise capacity and performance [7], this review instead describes the rationale and potential mechanistic basis for the proposed and emerging effects of EKS on recovery from exercise and adaptations to exercise training, as well as critically appraising the emerging literature in this field.

## 2 Metabolic and Molecular Effects of Ketone Bodies

Blood [KB] a typically ≤ 0.1 mM in the postprandial state, and ~0.1 to ~0.4 mM after an overnight fast [1, 2], and increases progressively to reach concentrations of ~4 mM after 7–10 days of fasting [46, 47]. βHB is a chiral molecule with two enantiomers, R‐ and S‐. R‐βHB is the circulating and primary form of βHB, with S‐βHB only contributing ~3% of [total βHB] even in individuals adhering to a ketogenic diet [48]. Circulating [R‐βHB] of ≥ 0.5 mM has been proposed as an operational definition of “nutritional ketosis” [3].

The main physiological role of the amplification of ketogenesis during low CHO availability is for KBs to replace glucose as the primary source of fuel for the brain, and to a lesser extent provide an additional substrate for other peripheral tissues such as heart and skeletal muscle [1, 2, 13]. Thus, AcAc and βHB have pleiotropic effects in multiple organs including brain, heart, and skeletal muscle by modulating substrate utilization, inflammation, oxidative stress, anabolic and catabolic processes, and gene expression [3, 13]. Many studies have used elevation of circulating [R‐βHB] via infusion as the exogenous means to demonstrate this range of metabolic actions [49], including attenuating glucose output by the liver [50, 51, 52] and glucose utilization in brain and skeletal muscle [52, 53], lowering circulating [FFA] [51, 52, 54] (likely through anti‐lipolytic effects on adipose tissue [55]), and attenuating proteolysis and stimulating muscle protein synthesis in skeletal muscle [50, 54, 56, 57, 58].

**FIGURE 1:**
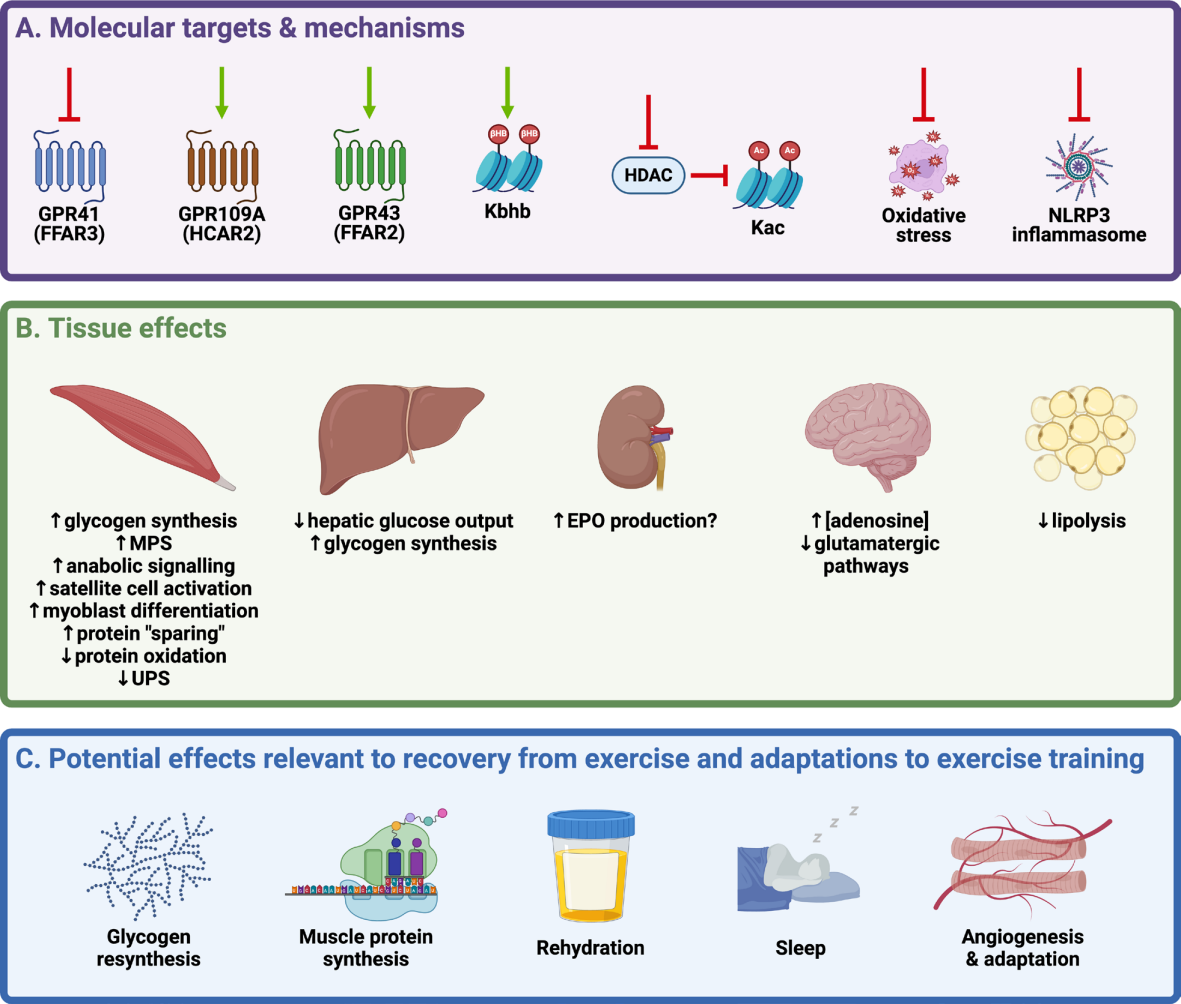
Molecular targets and tissue effects modulated by a change in KB concentration that are relevant to recovery from exercise and adaptations to exercise training. See text for abbreviations and detailed explanations. Created in BioRender.com.

Similarly, a range of effects on metabolism has been consistently observed after acute ingestion of EKS in various contexts both at rest, and during and after exercise [9, 22, 23, 27, 28, 32, 34, 37, 38, 59, 60, 61, 62, 63, 64, 65, 66, 67, 68, 69, 70, 71]. While these acute metabolic effects have been largely attributed to the action of KBs as substrates for metabolic pathways, AcAc and βHB are well‐established as signaling molecules capable of modulating an array of molecular events that regulate gene expression and related functional consequences [3, 13] (Figure 1). The precise pathways and potential for tissue‐specific regulation are subjects of ongoing investigation. One aspect of regulation is via G‐protein coupled receptor (GPCR) signaling, with R‐βHB being an endogenous ligand of GPR109A/HCAR2 [55] and GPR41/FFAR3 [72], and AcAc being an endogenous ligand of GPR43/FFAR2 [73]. For both KBs, the reported half‐maximal effective concentration (EC_50_), at least in adipocytes and HEK293 cells, is in the range of ~0.8–1.4 mM [55, 73], which is readily achievable for several hours after acute ingestion of EKS [7].

Additionally, whether via GPCR signaling at the cell membrane, or actions via changes in intracellular concentrations of KBs, a range of molecular pathways and processes that are modulated by KBs have been observed in preclinical models. These include regulation of gene expression through changes in histone lysine acetylation (Kac) via inhibition of histone deacetylases (HDACs) [74], and histone lysine β‐hydroxybutyrylation (Kbhb) [75], and modulation of the activity of the NLRP3 inflammasome [76] and the oxidative stress response [74]. Although these effects have been observed across tissues and cell types, few are confirmed as being activated in skeletal muscle, which is where much, although not all, of the focus for processes related to postexercise recovery and adaptations to exercise training tends to be.

However, some potentially relevant effects of KBs in skeletal muscle in vivo and in vitro include attenuation of the upregulation of the ubiquitin‐proteasome system (UPS) and autophagy‐lysosome pathways that are central to muscle atrophy during unloading [77], promotion of myoblast proliferation and differentiation in a GPR109A‐dependent manner [78], activation of skeletal muscle satellite cell proliferation and muscle regeneration after muscle injury [79], increased mitochondrial respiration, viability and fusion [80], increased insulin‐independent glucose uptake [81], and increased muscle protein synthesis (MPS) and activation of signaling downstream of mechanistic target of rapamycin complex 1 (mTORC1) [60].

## 3 Processes of Exercise Recovery and Training Adaptation: Potential Role for Addition of Exogenous Ketone Supplements

Many perturbations to homeostasis occur with the onset of, and recovery from, exercise, and are manifested in every organ system, but particularly so in the skeletal muscle being exercised [82]. Sports nutrition strategies often focus on optimizing recovery because nutrient‐responsive processes directly influence the ability to perform subsequent exercise, as well as augmenting cellular processes that impact adaptations to exercise training [83]. Given the waning interest and apparently limited value in the direct ergogenic effects of EKS [7], and because of the many and varied metabolic and molecular effects of KBs that overlap with processes that are central to exercise recovery and training adaptation, focus in recent years has turned to the potential role for EKS in recovery and adaptation [18].

One key aspect of recovery is the restoration of myocellular and whole‐body homeostasis in the immediate hours after exercise [84], and the replenishment of intramuscular substrates to pre‐exercise concentrations over 24–48 h after exercise [85, 86]. Consequently, appropriate nutrition strategies that restore fluid balance, augment glycogen resynthesis in liver and skeletal muscle, and stimulate MPS, are often given highest priority as part of the recovery process [85, 87, 88]. Appropriate quantity and quality of sleep are increasingly recognized as another important aspect of recovery for athletes [89], and may also be sensitive to nutrition intervention [90].

Restoration of fluid balance is dependent on the interaction between the volume of fluid ingested, the rate of fluid ingestion, and fluid composition employed in a postexercise rehydration strategy, as well as the time available to achieve euhydration [88]. The target volume to be ingested during recovery typically exceeds that lost as sweat during exercise (e.g., ingesting 150% of the body mass lost). The addition of sodium is beneficial due to its effect on extracellular fluid osmolality and volume, whereas the addition of CHO and protein can influence the absorption and distribution of ingested fluid, which in turn affects extracellular fluid osmolality and volume to promote the maintenance of euhydration [88].

The combination of CHO and protein is central to nutrition strategies that augment glycogen resynthesis and stimulate MPS [83, 85, 91]. Guidelines are well established for the type, timing and amount of CHO and protein to maximize muscle glycogen resynthesis and MPS in order to prepare for the next session of exercise or performance, and maximize anabolism to support adaptations to exercise training [83, 85, 91]. Ingestion of CHO at a rate of 1.2 g kg^−1^ h^−1^ during the first 4 h of recovery maximizes the rate of muscle glycogen resynthesis [87]. Addition of 0.4 g kg^−1^ h^−1^ protein added to “suboptimal” CHO doses during recovery can increase the rate of muscle glycogen resynthesis compared to suboptimal CHO alone [87]. When the time between exercise sessions or competition is limited (e.g., < 8 h), nutrition strategies to augment glycogen resynthesis for subsequent exercise performance are often considered as the highest priority [85]. However, when the period between sessions is longer (e.g., > 24 h), or if an athlete is deliberately targeting a period of low CHO availability [92], *maximizing* the rate of muscle glycogen resynthesis is of lower priority.

Another key aspect of recovery is the molecular and cellular contributions to exercise training‐induced adaptations [82]. The currently prevailing model proposes that factors intrinsic and extrinsic to muscle cells in exercising skeletal muscle are under the influence of neuronal, mechanical, metabolic, and hormonal stimuli that constitute perturbations to homeostasis. These stimuli, in turn, produce molecular signals that activate and/or repress signal transduction pathways and downstream effector proteins involved in the regulation of pre‐ and posttranscriptional processes, and of protein translation and degradation processes [82]. The phenotypic and functional consequences of exercise training are ultimately dependent on change at the protein level of key proteins involved in energy provision, the remodeling of cellular components such as contractile proteins and the extracellular matrix, and the biogenesis of organelles such as ribosomes and mitochondria; be that in the form of altered abundance, maximal activity, and/or sensitivity to the regulatory mechanisms determining activity or function [82]. Therefore, the process of adaptation to *any* type of exercise will require an increase in protein synthesis i.e., the synthesis of new proteins from existing, or acute exercise‐induced changes in, mRNA abundance.

The term MPS refers broadly to whole or ‘mixed’ muscle protein synthesis, but analyses of MPS at the level of bulk subfractions including myofibrillar (myoPS), sarcoplasmic (sarcPS) and mitochondrial (mitoPS) [93, 94, 95] have provided greater insight into the specifics of adaptive responses to different types of exercise. A working model is that acute aerobic and resistance exercise produce consequent effects to preferentially augment mitoPS and myoPS, respectively, and the cumulative effects over time are central to exercise training‐induced mitochondrial biogenesis [96] and myofibrillar protein accretion [97, 98], respectively. Thus, the dynamics of protein turnover, MPS and muscle protein breakdown (MPB), and proteostasis are central to adaptive responses to exercise training, with the regulation of MPS and MPB (degradation) becoming a central focus for understanding the mechanistic basis of adaptation [82]. Consequent to these cellular changes are alterations at the level of tissues and systems such as angiogenesis, muscle hypertrophy and altered substrate metabolism [99, 100, 101].

Notably, the activation of these molecular processes and the time course of recovery from a single session of exercise can be augmented or attenuated by the timing and type of nutrition provision in the postexercise period, and can influence the nature of molecular events during this time [85, 86, 97, 102]. A net positive balance whereby MPS exceeds MPB in the postexercise period can be augmented by ingestion of protein and CHO, through the combined impact of transient essential aminoacidemia (> ~1500 μM) to increase MPS, and modest insulinemia (~15–30 mU/L) to markedly attenuate MPB [97, 98]. The acute response of MPS to ingestion of a single protein bolus exhibits a dose–response relationship and is generally considered to be maximized at 20–30 g of protein [103, 104, 105]. However, a recent finding suggests that the time frame in which this effect is observed (typically 3–6 h) influences that conclusion, and that when the time frame is extended to 12 h, a bolus of 100 g produces a greater MPS response than 25 g [106]. Regardless, based on typical dietary intake patterns, athletes are currently advised to distribute protein intake evenly throughout the day (1.2–2.0 g kg^−1^ day^−1^) with each eating occasion, including a postexercise bolus, providing 0.24–0.40 g kg^−1^ of protein [107], although this dose may increase to 0.5 g kg^−1^ in endurance athletes, or when training with low CHO availability [108].

Given these established principles for recovery from exercise, if EKS are to be recommended as a central feature in strategies that enhance or optimize recovery, studies must demonstrate the effectiveness of EKS in combination with, or as an alternative to, current best practices for the numerous aspects of recovery and adaptation described above, including glycogen resynthesis, MPS, rehydration, sleep, and other molecular processes that underpin adaptive responses to exercise training.

## 4 Impact of Exogenous Ketone Supplements on Processes and Outcomes Relevant to Exercise Recovery and Training Adaptation

### 4.1 Glycogen Resynthesis in Skeletal Muscle

The first study to investigate the role of EKS on recovery from exercise provided 573 mg kg^−1^ of R‐BD R‐βHB KME immediately after a glycogen‐depleting bout of exercise (115 ± 2 min) alongside a 2 h 10 mM hyperglycaemic clamp [61]. Whole blood [R‐βHB] peaked at 5.3 ± 0.5 mM and remained elevated to 3.3 ± 0.2 mM at the end of the clamp period in the KME condition. Compared to the *non‐caloric* control (CON) condition, glucose uptake was increased by 32% during the clamp, and skeletal muscle glycogen concentration was ~50% greater in the R‐βHB KME condition when measured post‐clamp i.e., after 2 h of recovery. However, because glycogen was decreased to a greater extent after exercise in the CON (70 ± 23 mM kg dw^−1^) condition compared to KME (114 ± 23 mM kg dw^−1^), the calculated increase in glycogen concentration was 86% with KME (132 ± 20 mM kg dw^−1^) and 74% (94 ± 15 mM kg dw^−1^) with CON [61]. Therefore, the difference between conditions as relative change in skeletal muscle glycogen concentration was modest. A methodological caveat is that the 10 mM hyperglycaemic clamp is supraphysiological compared to studies of CHO alone or CHO‐protein co‐ingestion after glycogen‐depleting exercise, which generally do not observe circulating [glucose] of > 8 mM during the feeding period [60, 109, 110].

Two studies have since been performed that employed a recovery strategy with greater ecological validity, namely ingestion of drinks providing CHO and protein during recovery [60, 111]. The first of these studies provided 1.0 g kg^−1^ h^−1^ of CHO and 0.3 g kg^−1^ h^−1^ of whey protein hydrolysate in drinks consumed by 8 trained male participants for 5 h of recovery after a 90 min unilateral leg extension exercise protocol employed to deplete muscle glycogen (~60% to ~175 mM kg dw^−1^) [60]. This feeding strategy was chosen to elicit maximal rates of MPS and muscle glycogen resynthesis during the postexercise recovery period [85]. Participants also ingested 500 mg kg^−1^ of R‐BD R‐βHB KME immediately after exercise, followed by 250 mg kg^−1^ h^−1^ thereafter, which produced whole blood [R‐βHB] in the range of ~3–4 mM throughout the recovery period [60]. However, this postexercise ingestion of R‐BD R‐βHB KME did not augment muscle glycogen resynthesis compared to the control condition providing isocaloric long chain FAs [60].

The second study provided a recovery drink containing 1 g kg^−1^ of CHO and 0.4 g kg^−1^ of protein at 30 min after exercise to 9 recreationally active male participants who had completed 90 min of cycling at ~60%VO_2max_ [111]. R‐BD R‐βHB KME (1.25 g kg^−1^) was ingested in boluses of 500, 250, 250, and 250 mg kg^−1^ at min 45 of exercise, and at 0, 60, and 120 min after exercise respectively, resulting in serum [R‐βHB] in the range of ~3.3–4.7 mM throughout the recovery period. Compared to the taste‐matched placebo control condition, again postexercise ingestion of R‐BD R‐βHB KME did not result in differences between conditions in skeletal muscle glycogen concentrations when measured at 3 h after exercise [111].

Obvious differences in study design may explain the contrasting findings between the clamp study [61] compared to the latter two feeding studies [60, 111], including the different exercise modalities and magnitude of glycogen depletion, the different methods of increasing glucose availability i.e., nutrient provision, and the different durations of the recovery period. Also notable were differences in circulating [insulin], which was not different between conditions (~15 mU L^−1^) in one study using recovery drinks [60], but was two‐fold higher during the KME condition (KME: 31 ± 5 mU L^−1^; CON: 16 ± 3 mU L^−1^) in the clamp study [61], and was proposed as the mechanism for the augmented muscle glycogen resynthesis [61]. Conversely, early postexercise muscle glycogen resynthesis may be *independent* of circulating [insulin] when muscle glycogen concentrations are initially depleted below 150–200 mM kg dw^−1^ [87]. However, the effect of R‐βHB to enhance early postexercise muscle glycogen resynthesis independent of insulin is supported by a later study in mice [112]. In that study, male mice swam for 60 min after which the epitrochlearis muscle was excised and treated ex vivo for 2 h with either glucose (8 mM), insulin (60 mU L^−1^), or glucose and insulin plus 1, 2, or 4 mM of sodium R,S‐βHB. Muscle glycogen concentration was unchanged after treatment with 1 mM sodium R,S‐βHB, but was increased by ~15% and ~35% after treatment with 2 and 4 mM sodium R,S‐βHB, respectively [112]. This observation suggests a potential dose–response effect of βHB on skeletal muscle glycogen resynthesis, but a dose‐response effect is yet to be investigated in human participants.

The same research group followed that study with an investigation of changes in muscle glycogen in the 60 min after 60 min of treadmill running at 25 m min^−1^ when comparing postexercise oral administration of glucose and R‐BD R‐βHB KME to an isocaloric control of glucose and triolein [113]. The dose of R‐BD R‐βHB KME was 2.0 g kg^−1^, which was reported as equivalent to 162 mg kg^−1^ in humans. This dose produced a plasma [R‐βHB] in the range of ~2–3 mM, as well as a higher plasma [insulin], during the 60 min recovery period. Muscle glycogen concentrations after 60 min of recovery were not different between groups in the type II muscle fiber‐dominant plantaris muscle, but were 42% higher in the type I muscle fiber‐dominant soleus muscle in the R‐BD R‐βHB KME group [113]. Type I muscle fibers have a greater capacity to uptake and utilize KBs as a consequence of the greater protein content of monocarboxylate transporter 1 (MCT1), and greater enzymatic activity of the ketolytic enzymes 3‐hydroxybutyrate dehydrogenase (BDH), succinyl‐CoA:3‐oxoacid CoA transferase (OXCT), and acetyl‐CoA acetyltransferase (ACAT) [6], which may, in part, explain the greater response in the soleus muscle. The observation is also consistent with increased glucose conversion into glycogen in the soleus muscle, but not in the extensor digitorum longus muscle, another type II muscle fiber‐dominant muscle, previously observed in excised muscles from rats [114]. Whether any effect on glycogen resynthesis is time‐dependent warrants consideration because the greater glycogen resynthesis observed in a hyperglycaemic clamp [61], and in rodent experiments [112, 113], occurred over 1–2 h of recovery, whereas this glycogen resynthesis effect was not observed in the human studies investigating 3–5 h of recovery [60, 111].

A practical question relevant to situations of athletes training or competing more than once per day is whether, regardless of measurable effects on skeletal muscle glycogen, the addition of EKS to an optimal nutrition strategy impacts performance after short‐term recovery. To address this question, 9 males who participated in at least three endurance exercise training sessions per week completed a protocol involving a glycogen‐depleting interval exercise session lasting ~1 h followed by 4 h of recovery and subsequent performance of a 20 km TT, a 5 min recovery period and a 5 km TT [115]. The recovery strategy provided CHO drinks every 15 min for the initial 2 h of recovery in the form of a 10%–12% solution at a rate of 1.2 g kg^−1^ h^−1^, combined with either 25 g (347 ± 41 mg kg^−1^) of R‐BD R‐βHB KME or a dextrose placebo. Blood [R‐βHB] peaked at 2.6 ± 0.9 mM at 30 min and remained elevated at 1.2 ± 0.7 mM at 120 min. However, there were no significant differences between conditions in average power output, or time to complete the TTs (20 km TT: PLA, 38.86 ± 1.56 vs. KME, 38.38 ± 2.25 min; 5 km TT: PLA, 9.46 ± 0.88 vs. KME, 9.48 ± 0.65 min) [115].

In a similar study design, 13 endurance‐trained males completed ~50 min of high‐to‐moderate intensity treadmill running, which was followed by 4 h recovery before a treadmill run‐to‐exhaustion at ~70%VO_2peak_ [116]. The recovery strategy comprised of drinks every 30 min to provide CHO and protein at rates of 1.0 and 0.4 g kg^−1^ h^−1^, respectively. Participants also ingested either R‐BD R‐βHB KME at a rate of 290 mg kg^−1^ h^−1^, or an isocaloric olive oil placebo. Blood [R‐βHB] increased from ~1 mM at 30 min to ~4 mM at 4 h of recovery in KME, but no differences in plasma [insulin] were observed between KME and PLA [116]. Although muscle glycogen was not measured directly, breath samples were analyzed for CO_2_ production and ^13^C enrichment to determine the fate of ingested CHO, which demonstrated greater retention in KME (220 ± 26 g) compared to placebo (206 ± 26 g) (Hedge's *g* = 0.54). This modest difference was speculated to reflect net glycogen storage, which could have been in the liver, muscle, or both [116], but did not result in differences between conditions in the duration of the run‐to‐exhaustion (~55 min). In the KME condition, the blood [R‐βHB] was ~4 mM at the start of that run and remained elevated compared to PLA by ~1.2 mM at exhaustion, which somewhat confounds the interpretation of the run‐to‐exhaustion in the context of differences between conditions in the retention of ingested CHO [116].

### 4.2 Muscle Protein Synthesis and Associated Signaling Pathways

KBs are long established to exert an influence on muscle protein turnover through anabolic and anti‐catabolic processes. A 6 h infusion of sodium R,S‐βHB resulting in a circulating [R‐βHB] of ~2 mM produced a ~10% increase in MPS and a ~30% decrease in leucine oxidation in humans [57], whereas protein “sparing” has been observed when experimentally elevating KBs improves nitrogen balance (a proxy for protein turnover) under catabolic conditions including post‐surgery, skeletal trauma, severe burns, and sepsis [117]. Several studies have also provided mechanistic evidence for the anti‐catabolic effects of KBs [12].

The regulation of MPS and associated anabolic signaling pathways predominantly centers on the activation of canonical regulators of protein translation, namely mTORC1, S6K1, 4E‐BP1, and related parallel and downstream targets [82, 101]. In skeletal muscle, these anabolic pathways are responsive to changes in [KB] in vitro [60] and after ingestion of EKS [60]. When R‐BD R‐βHB KME was added to a CHO‐ and protein‐based recovery strategy producing a blood [R‐βHB] of ~3–4 mM ([60]; described in Section 4.1), greater phosphorylation of S6K1 and 4E‐BP1 was observed in human skeletal muscle biopsies after 5 h of recovery from a 90 min unilateral leg extension exercise protocol [60]. In the same study, the combination of 1.4 mM AcAc and 4 mM βHB augmented the phosphorylation of S6K1 and 4E‐BP1 in C2C12 myotubes in response to 1.5 mM leucine compared to 1.5 mM leucine alone [60]. This effect coincided with an approximately two‐fold greater MPS such that the 1.5 mM leucine plus KB condition was similar to 5.0 mM leucine alone [60].

Those data suggested that the addition of R‐BD R‐βHB KME augmented the response of anabolic signaling pathways and MPS to amino acid provision. This hypothesis was subsequently tested, albeit at rest rather than postexercise, by comparing the effects of R‐BD R‐βHB KME (360 mg kg^−1^) alone, 10 g whey protein alone, or their combination, on MPS (specifically myoPS), anabolic signaling and mTOR trafficking over a 5 h postprandial period in recreationally active young males [70, 118]. The protocol produced a blood [R‐βHB] of ~3 mM after 60 min in both conditions providing R‐BD R‐βHB KME, and remained above 1 mM at 180 min. As hypothesized, the ingestion of R‐BD R‐βHB KME alone stimulated an increase in MPS, but no differences were observed compared to whey protein alone or combined with R‐BD R‐βHB KME [70]. This finding contradicts the in vitro data that KBs potentiated MPS in leucine‐stimulated myotubes [60]. While there were no differences between conditions for phosphorylation of Akt, mTORC1, S6K1, or 4E‐BP1 [70], a follow‐up analysis revealed that combined R‐BD R‐βHB KME and whey protein resulted in a *sustained* increase in mTOR‐Rheb colocalization and translocation of mTORC1 toward the fiber periphery that was greater than either R‐BD R‐βHB KME or whey protein alone [118]. The functional relevance of this observation remains to be established, especially in the context of recovery from exercise given that this study was performed in the resting state rather than postexercise, employed a parallel group design, and the dose of protein was suboptimal compared to best practice guidelines for maximizing MPS.

The observations that KBs can exert both anabolic and anti‐catabolic effects in a range of contexts have led to the suggestion that EKS may support the maintenance of skeletal muscle mass in high‐stress environments [11]. Most human studies that inform this viewpoint have been acute in nature [56, 57, 58, 60, 70, 119]. The implication that EKS could have effects on skeletal muscle in the longer term relies on the principle that acute responses are predictive surrogate markers for those chronic effects, but this is not always the case with the MPS response and molecular signaling pathways [82].

One study has investigated whether intermittent exogenous ketosis through consumption of R‐BD R‐βHB KME (3 × 20 g daily) could impact changes in body composition including lean body mass (LBM) during 4 weeks of a hypocaloric (30% energy restriction) diet in female recreational athletes [120]. While a high protein condition (~2.1 g kg^−1^ day^−1^) mitigated the decline in LBM induced by the period of energy restriction, both the placebo and KME groups (protein intake ~1.0 g kg^−1^ day^−1^) experienced loss of LBM (~0.8 ± 0.2 kg), suggesting that daily intermittent exogenous ketosis did not provide anabolic and anti‐catabolic effects on skeletal muscle in this context. However, compared to placebo and high protein, the KME condition prevented the diet‐induced decline in resting energy expenditure, and mitigated the increase in perception of stress in those groups, as well as matching the effect of high protein to prevent the decline of 2.5% ± 0.7% in time to exhaustion during an incremental exercise test observed in the placebo group [120].

The effect of daily intermittent exogenous ketosis to blunt some of these negative effects corroborated an earlier study that investigated a 3‐week intervention that placed athletes at risk for excessive protein breakdown, inadequate recovery, and overreaching [121]. Physically active young males (*n* = 18) participated in an intensive cycling‐based training program incorporating constant‐load aerobic exercise training, intermittent endurance training, and high‐intensity intermittent exercise training, often twice daily for 6 days of each week. R‐BD R‐βHB KME was consumed two to three times daily (25 g in each dose) by being added to a CHO‐ and protein‐based recovery drink consumed after each training session, with an additional 25 g of R‐BD R‐βHB KME consumed prior to bedtime. Performance in a 30 min cycling TT performed during training, and in the week after training ceased, was not different between KME and control (medium‐chain triglycerides) conditions, but power output during a 30 min cycling TT performed after a 90 min pre‐load was ~15% greater with R‐BD R‐βHB KME compared to control on day 18 of the training program (KME: 216 ± 8 W; CON: 188 ± 14 W) [121]. Moreover, a range of other indicators of overreaching were attenuated including nocturnal urinary adrenaline excretion, resting heart rate, and submaximal and maximal heart rate responses to exercise. These data suggested that daily consumption of R‐BD R‐βHB KME blunted the symptoms of overreaching in this cohort. One outcome that impacts the interpretation of these findings is that important between‐group differences arose in dietary intake as a ‘spontaneous’ increase in energy intake in the KME group, which was evident as early as week 2 (~13%) of training and increased to ~20% in week 3 [121]. An increase in daily energy intake with daily consumption of R‐BD R‐βHB KME was also observed in a subsequent study simulating a 6 day endurance cycling race wherein average daily energy intake increased by ~700 kcal during the 6 day race period in the KME‐supplemented (1179 mg kg^−1^; ~11% of energy intake) group [122].

### 4.3 Exercise‐Induced Muscle Damage

Exercise‐induced muscle damage (EIMD) is a non‐pathological condition that arises following unaccustomed exercise, particularly when it involves repeated eccentric (i.e., lengthening) muscle contractions [123]. This form of exercise leads to skeletal muscle damage, typically marked by a rapid and prolonged (24–72 h) decline in muscle force, increased soreness, acute inflammation, and elevated concentrations in the blood of markers of muscle damage such as creatine kinase (CK) [123]. These physiological responses can temporarily impair muscle function and reduce the capacity to perform during subsequent exercise sessions, which has resulted in considerable interest in nutrition strategies that could ameliorate EIMD and associated outcomes, and/or accelerate recovery [124]. Because of a greater acute response of MPS to unaccustomed eccentric‐based exercise compared to a work‐matched session of concentric exercise [125, 126], recovery and successful remodeling of the damaged tissue have been proposed to depend on an increase in postexercise MPS to facilitate structural repair, while also limiting excessive MPB in the inflammatory environment. Yet, evidence for the efficacy of targeted protein intake to ameliorate EIMD is equivocal [124, 127]. Alternatively, KBs have pleiotropic roles in cellular metabolism with potential relevance to recovery from EIMD including effects on inflammation, oxidative stress, and skeletal muscle satellite cell activation [58, 74, 76, 79, 128, 129], which has resulted in investigations of the effects of EKS on recovery from EIMD [130, 131, 132].

The first of these studies employed a parallel group design in which participants ingested either R‐BD R‐βHB KME (360 mg kg^−1^ per dose), or isocaloric CHO, twice daily (morning and 30 min prior to bedtime) for 2 days after performing an exercise session consisting of 5 × 20 repetitions of 60 cm drop jumps to induce EIMD [130]. Supplementation commenced ~15 min after completion of the session, with acute ingestion producing whole blood [R‐βHB] of ~4.0 and ~4.1 mM at 30 and 60 min, respectively, after ingestion before a decline to ~1.3 mM at 180 min. However, between‐group differences were not evident at +24 or +48 h of recovery for muscle soreness, muscle function (maximum voluntary isometric contraction and countermovement jump), or markers of muscle damage and inflammation [130].

A second study employed a parallel group design in which participants ingested either R‐BD R‐βHB KME (27 g per dose), or isocaloric CHO, three times daily (9 a.m., 12 p.m., and 3 p.m.) for the 3 days after performing an exercise session consisting of 300 unilateral eccentric quadriceps contractions to induce EIMD [131]. Blood [R‐βHB] was only measured at time points that were ~3 h post‐ingestion so it provided limited insight into the response to each bolus, but based on the dose provided being similar to many previous studies, a reasonable assumption is that [R‐βHB] was elevated > 2 mM for as much as 6–9 h each day. However, between‐group differences were not evident at +3, +6, +24, +48 and +72 h of recovery for muscle soreness, muscle function (isokinetic work and isometric torque), or markers of muscle damage and inflammation [131].

Lastly, a third study employed a parallel group design in which participants ingested either R‐BD R‐βHB KME or non‐caloric placebo (CON) during an ultra‐endurance race and for 24 h of recovery after the race (running distance, 89 ± 15 and 80 ± 17 km for KME and CON; running time, 10.7 ± 2.4 and 9.7 ± 2.5 h for KME and CON) [132]. Provision of R‐BD R‐βHB KME produced blood [R‐βHB] of ~2 mM throughout the race, whereas additional boluses of 25 g were provided immediately postrace and 30 min before bedtime on race day, as well as 30 min before breakfast, lunch, and bedtime on the recovery day. Between‐group differences were not evident at +36 h of recovery for muscle soreness, muscle function (countermovement jump), or CK as a marker of muscle damage, but macrophage (CD68^+^) infiltration into skeletal muscle was prevented in KME, as opposed to an 80% increase in CON [132]. Whether macrophage infiltration at 36 h after exercise represents a pro‐ or an anti‐inflammatory response to exercise, especially given the arduous nature of ultra‐endurance [21], is unclear, so the relevance of this effect of KME acutely or in the longer term with repeated exercise sessions requires further investigation. Moreover, there is ambiguity about whether anti‐inflammatory interventions during recovery from exercise are beneficial or detrimental to adaptations to exercise training over the longer term [133].

### 4.4 Quality and Quantity of Sleep

Despite widespread recognition of the importance of quantity and quality of sleep in support of recovery from exercise, sleep disruption is common in athletes owing to in‐season variations in competition frequency and training load, and logistical factors such as travel requirements and evening competitions [89]. Sleep disruption in the aftermath of evening exercise is associated with worsened perceptual recovery, motivation to train, and next‐day exercise performance [134, 135, 136], whereas evening exercise itself, especially of high intensity, in close proximity to bedtime can negatively impact sleep quantity and quality [137]. There is much interest in nutrition interventions in this context given the obligatory requirement for nutrition as part of the recovery process after exercise [83], and because sleep metrics are sensitive to nutrition intervention [90].

To investigate the potential role of EKS to impact sleep after evening exercise, 10 well‐trained cyclists undertook a morning endurance training session (2 h), and an evening high‐intensity interval session (1.5 h) that ended 1 h before bedtime, while consuming 25 g of R‐BD R‐βHB KME (or placebo) immediately after each session, and 30 min before bedtime [66]. Analysis in a separate subset of participants suggested that this protocol would result in a blood [R‐βHB] of ~4 mM at sleep onset before declining to ~0.5 mM 5 h after sleep onset. The exercise stimulus resulted in disruptions to sleep as would be expected with such a protocol and timing [138], but KME counteracted the declines in sleep efficiency (the ratio of total sleep time to time in bed) and minutes of rapid eye movement (REM) sleep, as well as the increase in minutes of wakefulness after sleep onset [66]. These data suggest that the ingestion of R‐BD R‐βHB KME after evening high‐intensity exercise could mitigate disruptions to sleep efficiency and quality caused by that exercise. However, the magnitude of these differences warrants consideration for their practical relevance. For total sleep time, the difference between control (483 ± 42 min) and KME (499 ± 48 min) was only ~17 min, which represented a *trivial* effect size (Cohen's *d* = 0.05), whereas this difference when expressed as wakefulness after sleep onset represented a reduction of almost a half with KME (20 ± 15 min) compared to control (37 ± 20 min) (*moderate* effect size; Cohen's *d* = 0.62). Given that the study design employed a 90 min session of high‐intensity interval exercise just 1 h before bedtime i.e., effectively after dinner and without an appropriate recovery meal, the ecological validity of this study design also was arguably low, and could be improved in future studies.

Another stimulus resulting in sleep disruption is hypoxia to which athletes are exposed during altitude training camps [139]. Using a train low‐live high model wherein athletes performed two exercise sessions during ~8 h at sea level, but moved to and slept at simulated 3000 m over the next ~16 h, ingestion of R‐BD R‐βHB KME (4 × 25 g) throughout the day and before bedtime did not counteract the declines in sleep efficiency and minutes of slow‐wave sleep induced by hypoxic exposure [140]. Beyond exercise, there are very few studies examining the impact of KBs on sleep. Of note, however, is that intracerebroventricular injection of AcAc (but not βHB) increased slow wave during non‐REM sleep in a dose‐dependent manner in mice [141], whereas 2 weeks of daily consumption of an R‐βHB acid supplement (low, 1.5 g R‐βHB; high 2.9 g R‐βHB) improved various subjective measures of sleep in middle‐age and older adults who were experiencing temporary fatigue and poor sleep quality [142].

Although neuronal and glial cells of the brain are avid users of KBs when they are abundant as a substrate [16], the mechanisms by which ingestion of EKS impacts sleep are largely speculative at present, and are often extrapolated from studies of ketogenic diets [18, 143]. These mechanisms include the potential that inhibition of adenosine kinase activity by KBs increases extracellular [adenosine] [144], or that the metabolism of βHB alters the concentration of TCA cycle intermediaries and reduces glutamate transamination, instead favoring γ‐aminobutyric acid (GABA) synthesis [145, 146]. An increase in adenosine could increase homeostatic sleep pressure and promote slow‐wave sleep, whereas the inhibition of glutamatergic pathways could suppress neuronal excitability and shift brain activity toward an inhibitory state that is conducive to sleep onset [147].

### 4.5 Restoration of Fluid Balance After Exercise‐Induced Dehydration

One of the important effects of the addition of sodium to a rehydration solution is to reduce urine output during the recovery period after exercise‐induced dehydration such that more of the ingested fluid is retained and net fluid balance is achieved [148, 149]. The effect of the ingestion of EKS during recovery from exercise on the restoration of fluid balance has not yet been reported, but several studies have consistently observed attenuated urine production *during* exercise when EKS are ingested before and during exercise [9, 23, 64, 150].

Each study followed a largely similar protocol in which trained cyclists completed a simulated cycling race consisting of 3 h of intermittent intensity cycling exercise, a 15 min cycling TT, and a maximal sprint at 175% of lactate threshold. Ingestion of R‐BD R‐βHB KME was 65 g as 25 g, 20 g, and 20 g boluses at 60 min prior to, 20 min prior to, and 30 min into, the intermittent intensity cycling period [9, 23, 150], and 75 g as 25 g boluses at 30, 90, and 150 min into the intermittent intensity cycling period [64]. These dosing strategies increased circulating [R‐βHB] to ~2–3 mM during exercise. Urine output collected during the exercise trials was ~43% [9], ~32% [23], ~18% [64], and ~20% [150] lower in KME compared to control conditions. In one study, total urine output was observed to have a moderate negative correlation with circulating [R‐βHB] (*r* = −0.69) [150]. The approximately 20%–40% reductions in urine output across those four studies were only ~220 mL in absolute terms over the ~3.5 h period, yet are indicative of an antidiuretic effect of intermittent exogenous ketosis, at least *during* exercise. Only one of the studies to date explored the mechanistic basis of this reduction in urine output, and suggested that neither aldosterone nor vasopressin were involved in this reduction [150].

Further investigation of regulatory mechanisms and whether the antidiuretic effect is observed during postexercise rehydration is warranted because ingestion of large volumes of fluid in a short period of time stimulates diuresis even in the hypohydrated state [88]. Therefore, an antidiuretic effect of EKS would be most relevant to postexercise rehydration in conditions when the rehydration strategy is aggressive and the recovery period is short e.g., < 8 h. Under resting conditions, one study observed no effect of either R,S‐βHB salts and R‐BD R‐βHB KME on urine output over 4 h post‐ingestion, albeit there was limited detail reported [59]. Another study observed no difference between R‐BD R‐βHB KME and placebo (729 ± 342 vs. 729 ± 248 mL, respectively) when urine was collected throughout the night in the aforementioned study of R‐BD R‐βHB KME being consumed after exercise performed 1 h before bedtime [66]. Lastly, during a 4 h recovery period after ~50 min of treadmill running in endurance‐trained males, *voluntary* fluid intake was ~9% lower in R‐BD R‐βHB KME compared to placebo (2835 ± 455 vs. 3116 ± 538 mL), and urine output was also ~13% lower in R‐BD R‐βHB KME (1186 ± 499 vs. 1359 ± 410 mL) [116]. Calculated as a percentage of fluid ingested, urine output was ~42% and ~44% in the R‐BD R‐βHB KME and placebo conditions, respectively, suggesting little effect of R‐BD R‐βHB KME ingestion on urine output during postexercise recovery [116]. Net fluid balance was not measured in that study, but if intermittent exogenous ketosis has the effect to reduce thirst and urge to drink, akin to its effects on appetite [63, 151], the overall effect during recovery from exercise could be impaired rather than improved rehydration outcomes.

### 4.6 Elevated Erythropoietin (EPO) Concentrations and Angiogenesis in Skeletal Muscle

Studies employing administration of KBs via intravenous infusion [49] have informed much of the development and application of EKS in athletic contexts by providing insights into potential mechanisms and efficacy [7]. Another example is the observation of an increase in circulating [EPO] in response to infusion of sodium R‐βHB [152]. In two studies combining 17 middle‐aged male and female participants, infusion of sodium R‐βHB resulted in circulating [R‐βHB] of ~3.8 and ~5.5 mM after 390 and 240 min, respectively, of infusion. Compared to a saline control, this hyperketonemia resulted in ~29% higher serum [EPO] (9.9 ± 1.1 vs. 7.6 ± 1.0 IU L^−1^) [152]. The mechanism by which the stimulation of EPO occurs remains elusive, but these results have been partially corroborated by findings of an increase in circulating [EPO] under the influence of acute [67] or daily [153, 154] ingestion of EKS.

Specific to acute exercise and recovery, 9 healthy males completed a 1 h session of cycling intervals after which they ingested 0.8 g kg^−1^ of CHO and 0.4 g kg^−1^ of protein at 0, 1, 2, and 3 h of recovery, with or without 290 mg kg^−1^ of R‐BD R‐βHB KME ingested at 0, 1, and 2 h of recovery [67]. Serum [R‐βHB] increased by > 1 mM within 15 min and reached a peak of 3.2 ± 0.5 mM at 180 min. At the end of the 4 h recovery period, serum [EPO] was ∼20% higher in KME compared to control, with the iAUC over 4 h for serum [EPO] being ~3‐fold greater in KME. However, a later study of 9 recreationally active males who completed 90 min of cycling at ~60%VO_2max_ observed no increase in serum [EPO] in either R‐BD R‐βHB KME or control conditions after a 3 h recovery period [111]. The authors suggested that longer exposure to elevated [R‐βHB] may be needed to elicit an increase in serum [EPO] because other studies had utilized 4–7 h of elevated [R‐βHB] [67, 152], but this contention is contradicted by an increase in serum [EPO] being observed at 2 and 3 h postexercise in one of those studies [67]. More relevant perhaps is that serum [EPO] was consistently higher at rest, and during exercise and recovery (~10–13 IU L^−1^) in that most recent study [111] compared to the earlier studies [67, 152]. Those higher values were greater than the highest values achieved in KME conditions in those earlier studies i.e., 9.9 ± 1.1 IU L^−1^ [152] and 9.0 ± 2.3 IU L^−1^ [67], which may have resulted in there being a limited range for an increase in serum [EPO] in response to acute exercise or exogenous ketosis. Another study reported no difference between KME and control for the increase in serum [EPO] in samples taken *immediately after* a 6.4 km load carriage time trial (~55 min) [155], but that observation was not surprising because previous studies observing an effect on [EPO] have involved higher [R‐βHB] and longer duration of postexercise exposure to elevated [R‐βHB] [67, 152].

If a postexercise R‐βHB‐induced increase in serum [EPO] is replicated in future studies, the functional consequence of such an effect would be of potential interest to athletes. EPO stimulates erythropoiesis by the kidneys, and thereby increases red blood cell number, hemoglobin mass and oxygen‐carrying capacity of blood [156], a critical factor in maximal effort endurance exercise performance [157]. Nutrition (e.g., cobalt supplementation) and training (e.g., altitude camps) strategies that seek to increase erythropoiesis and hemoglobin mass are, therefore, undertaken by athletes [139, 158]. The magnitude of postexercise increase in circulating [EPO] when R‐BD R‐βHB KME was ingested is similar to that of cobalt supplementation and altitude exposure [139, 158], which suggests that intermittent exogenous ketosis after individual exercise sessions could augment changes in erythropoiesis and hemoglobin mass in response to exercise training. That hypothesis remains to be tested. Importantly in this context, the magnitude of increase in erythropoiesis in response to administration of recombinant human EPO is primarily determined by the length of time that an elevated [EPO] is maintained [159], so repeated ingestion of EKS throughout the day is likely to be necessary to elicit an erythropoietic effect.

A secondary analysis of the 3 week aerobic training overreaching study with daily ingestion of R‐BD R‐βHB KME described in Section 4.2 [121] revealed a ~25% increase in [EPO] in blood samples taken 10–13 h after the last exercise session [153]. Whether this increase was an effect of the dose of R‐BD R‐βHB KME ingested after that last training session, or an effect of exercise training combined with KME, was not possible to distinguish. Unfortunately, other hematological measures were not measured, so the effects on hemoglobin mass and hematocrit are unknown. However, that study did analyze skeletal muscle biopsies for markers of angiogenesis and its regulation [153]. Such an analysis is salient because positive effects of EPO on skeletal muscle, as distinct from hematological effects, have been hypothesized [156], and skeletal muscle angiogenesis is one of the hallmarks of adaptation to aerobic exercise training [82]. After the 3 week intervention, the number of capillary contacts and the capillary‐to‐fiber perimeter exchange index measured by immunohistochemistry were increased by 44% and 42%, respectively, in the KME group. mRNA and protein abundance of vascular endothelial growth factor (VEGF), a key molecular regulator of angiogenesis, were also increased, such that the %changes in [EPO] and VEGF protein abundance were positively correlated (*r* = 0.749) [153]. One interpretation of these data is that a KME‐induced repeated daily increase in [EPO] resulted in the stimulation of VEGF activity and skeletal muscle angiogenesis, but there are a number of caveats to that interpretation [160]. Firstly, there was no evidence of the expected increase in angiogenesis in the control condition. Secondly, as described in the parent study [121], the KME condition resulted in ~15% greater training volume in the third week, and whereas an energy deficit emerged in the control condition, energy intake in the KME condition increased to match energy demands and maintain energy balance. Together, these caveats suggest that energy deficit may have contributed to the lack of positive adaptation in the control condition, with the overreaching stimulus having a suppressive effect on the normal angiogenic response to exercise training, and this suppression being inhibited in the KME condition. Lastly, there is some doubt as to whether EPO has direct effects on skeletal muscle given that it remains to be confirmed that the EPO receptor in skeletal muscle is functionally responsive to physiological concentrations of EPO [161]. Nevertheless, these data warrant further investigation of how daily postexercise ingestion of R‐BD R‐βHB KME (or other EKS) could impact adaptations to exercise training. The relative ease with which intermittent exogenous ketosis can be achieved daily for extended periods has expanded the potential utility of EKS in diverse settings for mitigation of skeletal muscle atrophy [11, 12], which for athletes, beyond intensified training, could also include phases of intentional weight loss via energy restriction such as in weight category sports, and periods of forced inactivity due to injury.

Lastly, as a signaling molecule, R‐βHB has been demonstrated to regulate gene expression through changes in histone Kac as a HDAC inhibitor [74], and through histone Kbhb [75] (Figure 1). Such effects could, in theory, produce changes in molecular pathways that would overlap with those described in skeletal muscle after exercise [82], which suggests that postexercise ingestion of EKS could modulate the molecular response to acute exercise. Only one study, to date, has investigated this question with an omics approach, which was a transcriptomic analysis of skeletal muscle biopsy samples taken 3 h after 90 min of cycling at ~60%VO_2max_ [111]. R‐BD R‐βHB KME (1.25 g kg^−1^) ingestion during and after exercise resulted in serum [R‐βHB] in the range of ~3.3–4.7 mM throughout the recovery period. Of 16 898 genes measured, 1561 were differentially expressed (909 increased, 652 decreased) when using a false discovery rate of < 0.05 and a fold change cutoff of ±1.5. Yet despite this clear exercise effect on the skeletal muscle transcriptome, there was no effect of condition, nor was any individual gene differentially expressed after R‐BD R‐βHB KME ingestion [111].

## 5 Perspective and Conclusion

In contrast to the inconsistent and underwhelming evidence for acute ingestion of EKS as a direct ergogenic aid [7], the studies described in this review point to several possible avenues in exercise recovery and training adaptation. These findings may partly explain the anecdotally reported increase in the use of EKS for recovery in elite endurance sports such as professional cycling [162, 163]. Based on the protocols described in the published research to date, the cost of supplementation is likely to present a barrier to widespread use beyond professional sports in which greater budgets are available. For example, in Q3 2025 the R‐BD R‐βHB KME retails at ~$25 to $35 per 25 to 30 g of R‐BD R‐βHB KME (e.g., KE4, KetoneAid; Ketone Performance, deltaG), which translates to > $50 after *each* training session to support the recovery and adaptive processes described to date [60, 61, 66, 67, 121, 122, 153]. R,S‐βHB and R‐βHB salts, 1,3‐butanediol (e.g., KetoneIQ, HMVN) and newly developed ketone esters (e.g., Bis‐octanoyl (R)‐1,3‐butanediol ketone diester, Qitone) (Table 1) are less costly per individual dose, but remain to be tested for their effectiveness in supporting recovery from exercise. When R,S‐βHB salts and 1,3‐butanediol are ingested before and during exercise, increases in circulating [R‐βHB] are modest (generally < 1.0 mM) under the dosing strategies employed to date [7]. However, given that the oxidation of KBs by exercising muscle and other tissues is elevated during exercise [2], and thereby clearance of KBs is increased [22, 164, 165], ingestion of ketone salts and ketogenic precursors such as 1,3‐butanediol may produce larger increases in circulating [R‐βHB] per dose consumed under resting or postexercise conditions compared to ingestion before and during exercise. Such an effect would create a better rationale for these types of EKS in supporting recovery rather than acute exercise performance.

Dose‐dependent effects have been observed on various aspects of physiology and metabolism at rest and during exercise, whether by infusion of KBs, or ingestion of EKS [31, 45, 52, 164, 166, 167], yet absent to date is the investigation of whether any of the responses relevant to recovery from exercise are dose‐dependent. Most studies that have focused on recovery and adaptation have used dosing strategies that produced [R‐βHB] in a range of 2–5 mM. In that context, R‐βHB has an EC_50_ of ~0.8 mM as an endogenous ligand of the GPR109A receptor in adipocytes [55], and inhibition of histone deacetylases (HDAC) 1, 3, and 4 is evident in a dose‐dependent manner beginning at 1 mM in HEK293 cells [74]. Moreover, in vivo infusion of R‐βHB in healthy young males to a concentration of as little as ~0.2–0.5 mM elicits changes in whole‐body metabolism including attenuation of estimated hepatic glucose output and adipose tissue lipolysis, and increases in cerebral R‐βHB uptake [52]. Therefore, if the effects of EKS continue to show promise as beneficial for recovery from exercise, further exploration of the effects of different doses and types of EKS will be important because the circulating [R‐βHB] is likely to be an important determinant of the metabolic and molecular consequences of intermittent exogenous ketosis.

In conclusion, a rich history of preclinical models and human infusion studies clearly demonstrates that AcAc and R‐βHB have wide‐ranging metabolic and molecular effects on multiple organs, many of which can be recapitulated by acute ingestion of EKS, and many of which have a theoretical rationale for potential benefit for recovery from exercise and adaptation to exercise training. To date, studies suggest no benefit for recovery from EIMD, but potential benefits around muscle glycogen resynthesis and anabolic response during recovery, and mitigation of the negative impact of intense exercise on sleep (acutely) and short‐term overreaching, yet studies on these topics remain small in number, and several important knowledge gaps remain (Table 2). Whether there is any utility of EKS to the broader active adult population, rather than more specifically to highly trained and elite athletes, is unknown. Wider recommendations for EKS as an evidence‐based support for recovery and adaptation will require studies that ideally employ designs that are ecologically valid and that demonstrate the effectiveness of EKS in combination with, or as an alternative to, current best practices for the numerous aspects of recovery and adaptation, including sleep, rehydration, glycogen resynthesis, MPS, and other molecular processes that underpin adaptive responses to exercise training.

**TABLE 2:** Summary of current research and knowledge gaps in the application of intermittent exogenous ketosis to recovery from exercise and adaptations to exercise training.

Outcome	Summary of current research	Knowledge gaps to address with future studies^a^
Postexercise glycogen resynthesis in skeletal muscle	Greater glycogen resynthesis for 1–2 h of recovery observed in a hyperglycaemic clamp [61], and in rodent experiments [112, 113], but this effect was not observed in human studies with CHO‐protein feeding over 3–5 h of recovery [60, 111] No effect on subsequent cycling TT [115] or running TTE [116] when R‐BD R‐βHB KME was ingested during a 4 h recovery period after glycogen‐depleting exercise	Are effects, if any, of IEK on muscle (or liver) glycogen resynthesis dose‐, muscle fiber type‐ or time‐dependent, including ≥ 4 h and ≤ 24 h i.e., two‐a‐day training or next day performance?
Muscle protein synthesis and associated signaling pathways	Activation of anabolic signaling proteins after ingestion of R‐BD R‐βHB KME [60, 70, 118], and treatment of skeletal muscle cells with KBs [60] Anti‐catabolic effects of KBs observed in various experimental models [12, 117], but daily R‐BD R‐βHB KME ingestion did not attenuate LBM lost during 4 weeks of energy restriction [120] Daily R‐BD R‐βHB KME ingestion blunted the symptoms of overreaching during 3 weeks of intensified training [121]	Does postexercise ingestion of EKS augment anabolic signaling and MPS in response to exercise? Does postexercise ingestion of EKS alter the molecular response to exercise and augment adaptive responses to exercise training? Are potential anti‐catabolic effects of IEK relevant to improving outcomes in situations such as overreaching, injury, surgery, and weight loss?
Exercise‐induced muscle damage	No effect of IEK compared to control condition on muscle soreness, recovery of muscle function, or markers of muscle damage and inflammation [130, 131, 132]	Null effects observed in 3 studies to date, and absence of mechanistic rationale for effects of IEK on EIMD or DOMS, suggests that the justification for additional studies focussed on these outcomes is weak
Quality and quantity of sleep	One study observed small effects of R‐BD R‐βHB KME ingested before bedtime to counteract negative effects of late evening exercise on sleep quantity and quality [66]	Are effects of IEK on sleep quantity and quality observed in study designs that are more ecologically‐valid? Does ingestion of EKS before bedtime in the absence of disruption by late evening exercise positively impact sleep?
Restoration of fluid balance after exercise‐induced dehydration	Urine output *during* exercise is attenuated after ingestion of R‐BD R‐βHB KME (observed in 4 studies) [9, 23, 64, 150] Postexercise rehydration has not been directly studied in designs with matched fluid intake, but in one study postexercise ad libitum fluid intake was lower after ingestion of R‐BD R‐βHB KME [116]	Does ingestion of EKS, when combined with best‐practice fluid and electrolyte intake, result in reduced urine output or better fluid balance compared to current best‐practices alone?
Elevated EPO concentrations and angiogenesis in skeletal muscle	Elevated serum [EPO] after IEK observed in 2 studies [67, 152], but not observed in 2 other studies [111, 155], although important details differ between the study designs Greater angiogenesis after exercise training undertaken with repeated postexercise EKS ingestion [153]	Is the postexercise increase in serum [EPO] after ingestion of EKS during recovery consistently observed? If repeated throughout training, does this potential postexercise increase in serum [EPO] augment angiogensis and other adaptive responses to exercise training?

Abbreviations: DOMS, delayed‐onset muscle soreness; EIMD, exercise‐induced muscle damage; EKS, exogenous ketone supplements; EPO, erythropoietin; IEK, intermittent exogenous ketosis; KBs, ketone bodies; MPS, muscle protein synthesis; R‐BD R‐βHB KME, (R)‐3‐hydroxybutyl (R)‐3‐hydroxybutyrate ketone monoester; TT, time trial; TTE, time‐to‐exhaustion.

^a^ Assumes that the comparator condition is an isocaloric, taste‐matched placebo, with experiments performed as double‐blind whenever possible.

## Author Contributions

B.E. conceptualized, wrote and finalized the article.

## Ethics Statement

The author has nothing to report.

## Consent

The author has nothing to report.

## Conflicts of Interest

The author declares no conflicts of interest.

## Data Availability

Data sharing not applicable to this article as no datasets were generated or analyzed during the current study.

## References

[1] A. M. Robinson and D. H. Williamson , “Physiological Roles of Ketone Bodies as Substrates and Signals in Mammalian Tissues,” Physiological Reviews 60 (1980): 143–187. 6986618 10.1152/physrev.1980.60.1.143

[2] E. O. Balasse and F. Fery , “Ketone Body Production and Disposal: Effects of Fasting, Diabetes, and Exercise,” Diabetes/Metabolism Reviews 5 (1989): 247–270. 2656155 10.1002/dmr.5610050304

[3] A. M. Poff , A. P. Koutnik , and B. Egan , “Nutritional Ketosis With Ketogenic Diets or Exogenous Ketones: Features, Convergence, and Divergence,” Current Sports Medicine Reports 19 (2020): 251–259. 32692060 10.1249/JSR.0000000000000732

[4] H. Brunengraber , “Potential of Ketone Body Esters for Parenteral and Oral Nutrition,” Nutrition 13 (1997): 233–235. 9131688 10.1016/s0899-9007(96)00409-1

[5] R. L. Veech , “The Therapeutic Implications of Ketone Bodies: The Effects of Ketone Bodies in Pathological Conditions: Ketosis, Ketogenic Diet, Redox States, Insulin Resistance, and Mitochondrial Metabolism,” Prostaglandins, Leukotrienes, and Essential Fatty Acids 70 (2004): 309–319. 14769489 10.1016/j.plefa.2003.09.007

[6] M. Evans , K. E. Cogan , and B. Egan , “Metabolism of Ketone Bodies During Exercise and Training: Physiological Basis for Exogenous Supplementation,” Journal of Physiology 595 (2017): 2857–2871. 27861911 10.1113/JP273185 PMC5407977

[7] M. Evans , T. S. McClure , A. P. Koutnik , and B. Egan , “Exogenous Ketone Supplements in Athletic Contexts: Past, Present, and Future,” Sports Medicine 52 (2022): 25–67. 10.1007/s40279-022-01756-2 PMC9734240 36214993

[8] P. J. Cox and K. Clarke , “Acute Nutritional Ketosis: Implications for Exercise Performance and Metabolism,” Extreme Physiology & Medicine 3 (2014): 17. 25379174 10.1186/2046-7648-3-17 PMC4212585

[9] C. Poffé , M. Ramaekers , S. Bogaerts , and P. Hespel , “Exogenous Ketosis Impacts Neither Performance nor Muscle Glycogen Breakdown in Prolonged Endurance Exercise,” Journal of Applied Physiology (1985) 128 (2020): 1643–1653. 10.1152/japplphysiol.00092.2020 PMC7311686 32407242

[10] C. Poffé and P. Hespel , “Ketone Bodies: Beyond Their Role as a Potential Energy Substrate in Exercise,” Journal of Physiology 598 (2020): 4749–4750. 32969026 10.1113/JP280597

[11] B. J. Stubbs , A. P. Koutnik , J. S. Volek , and J. C. Newman , “From Bedside to Battlefield: Intersection of Ketone Body Mechanisms in Geroscience With Military Resilience,” Geroscience 43 (2021): 1071–1081. 33006708 10.1007/s11357-020-00277-y PMC8190215

[12] A. P. Koutnik , D. P. D'Agostino , and B. Egan , “Anticatabolic Effects of Ketone Bodies in Skeletal Muscle,” Trends in Endocrinology and Metabolism 30 (2019): 227–229. 30712977 10.1016/j.tem.2019.01.006

[13] P. Puchalska and P. A. Crawford , “Metabolic and Signaling Roles of Ketone Bodies in Health and Disease,” Annual Review of Nutrition 41 (2021): 49–77. 10.1146/annurev-nutr-111120-111518 PMC8922216 34633859

[14] S. A. Daines , “The Therapeutic Potential and Limitations of Ketones in Traumatic Brain Injury,” Frontiers in Neurology 12 (2021): 723148. 34777197 10.3389/fneur.2021.723148 PMC8579274

[15] S. R. Yurista , C. T. Nguyen , A. Rosenzweig , R. A. de Boer , and B. D. Westenbrink , “Ketone Bodies for the Failing Heart: Fuels That Can Fix the Engine?,” Trends in Endocrinology and Metabolism 32 (2021): 814–826. 34456121 10.1016/j.tem.2021.07.006

[16] É. Myette‐Côté , A. Soto‐Mota , and S. C. Cunnane , “Ketones: Potential to Achieve Brain Energy Rescue and Sustain Cognitive Health During Ageing,” British Journal of Nutrition 128 (2022): 407–423. 34581265 10.1017/S0007114521003883

[17] K. Falkenhain , A. Daraei , S. C. Forbes , and J. P. Little , “Effects of Exogenous Ketone Supplementation on Blood Glucose: A Systematic Review and Meta‐Analysis,” Advances in Nutrition 13 (2022): 1697–1714. 35380602 10.1093/advances/nmac036 PMC9526861

[18] R. Robberechts and C. Poffé , “Defining Ketone Supplementation: The Evolving Evidence for Postexercise Ketone Supplementation to Improve Recovery and Adaptation to Exercise,” American Journal of Physiology. Cell Physiology 326 (2024): C143–c160. 37982172 10.1152/ajpcell.00485.2023

[19] S. E. Deemer , B. M. Roberts , D. L. Smith , E. P. Plaisance , and A. Philp , “Exogenous Ketone Esters as a Potential Therapeutic for Treatment of Sarcopenic Obesity,” American Journal of Physiology. Cell Physiology 327 (2024): C140–c150. 38766768 10.1152/ajpcell.00471.2023

[20] S. Soni , R. J. Skow , S. Foulkes , M. J. Haykowsky , and J. R. B. Dyck , “Therapeutic Potential of Ketone Bodies on Exercise Intolerance in Heart Failure: Looking Beyond the Heart,” Cardiovascular Research 121 (2025): 230–240. 39825790 10.1093/cvr/cvaf004 PMC12012446

[21] L. Engelbrecht , E. Terblanche , K. Koppo , and C. Poffé , “Can Endogenous or Exogenous Ketosis Tackle the Constraints of Ultraendurance Exercise?,” Exercise and Sport Sciences Reviews 53 (2025): 60–67. 39680510 10.1249/JES.0000000000000357

[22] P. J. Cox , T. Kirk , T. Ashmore , et al., “Nutritional Ketosis Alters Fuel Preference and Thereby Endurance Performance in Athletes,” Cell Metabolism 24 (2016): 256–268. 27475046 10.1016/j.cmet.2016.07.010

[23] C. Poffé , M. Ramaekers , S. Bogaerts , and P. Hespel , “Bicarbonate Unlocks the Ergogenic Action of Ketone Monoester Intake in Endurance Exercise,” Medicine and Science in Sports and Exercise 53 (2021): 431–441. 32735112 10.1249/MSS.0000000000002467 PMC7803447

[24] O. J. Peacock , J. T. Gonzalez , S. P. Roberts , A. Smith , S. Drawer , and K. A. Stokes , “Ketone Monoester Ingestion Alters Metabolism and Simulated Rugby Performance in Professional Players,” International Journal of Sport Nutrition and Exercise Metabolism 32 (2022): 334–341. 35487576 10.1123/ijsnem.2021-0346

[25] D. J. Ramos‐Campo , F. J. López‐Román , S. Pérez‐Piñero , et al., “Effects of Ketone Monoester and Bicarbonate Co‐Ingestion on Cycling Performance in WorldTour Cyclists,” International Journal of Sport Nutrition and Exercise Metabolism 34 (2024): 1–10. 37751902 10.1123/ijsnem.2023-0078

[26] S. Rodger , D. Plews , P. Laursen , and M. W. Driller , “Oral β‐Hydroxybutyrate Salt Fails to Improve 4‐Minute Cycling Performance Following Submaximal Exercise,” Journal of Science and Cycling 6 (2017): 26–31.

[27] M. Evans and B. Egan , “Intermittent Running and Cognitive Performance After Ketone Ester Ingestion,” Medicine and Science in Sports and Exercise 50 (2018): 2330–2338. 29944604 10.1249/MSS.0000000000001700

[28] M. Evans , F. T. McSwiney , A. J. Brady , and B. Egan , “No Benefit of Ingestion of a Ketone Monoester Supplement on 10‐Km Running Performance,” Medicine and Science in Sports and Exercise 51 (2019): 2506–2515. 31730565 10.1249/MSS.0000000000002065

[29] B. E. Scott , P. B. Laursen , L. J. James , et al., “The Effect of 1,3‐Butanediol and Carbohydrate Supplementation on Running Performance,” Journal of Science and Medicine in Sport 22 (2019): 702–706. 30553764 10.1016/j.jsams.2018.11.027

[30] D. M. Shaw , F. Merien , A. Braakhuis , D. Plews , P. Laursen , and D. K. Dulson , “The Effect of 1,3‐Butanediol on Cycling Time‐Trial Performance,” International Journal of Sport Nutrition and Exercise Metabolism 29 (2019): 466–473. 30632425 10.1123/ijsnem.2018-0284

[31] P. J. Prins , D. P. D'Agostino , C. Q. Rogers , et al., “Dose Response of a Novel Exogenous Ketone Supplement on Physiological, Perceptual and Performance Parameters,” Nutrition & Metabolism (London) 17 (2020): 81. 10.1186/s12986-020-00497-1 PMC7523040 33005207

[32] D. G. McCarthy , W. Bostad , F. J. Powley , J. P. Little , D. L. Richards , and M. J. Gibala , “Increased Cardiorespiratory Stress During Submaximal Cycling After Ketone Monoester Ingestion in Endurance‐Trained Adults,” Applied Physiology, Nutrition, and Metabolism 46 (2021): 986–993. 10.1139/apnm-2020-0999 33646860

[33] M. D. Quinones and P. W. R. Lemon , “Acute Ketone Salts‐Caffeine‐Taurine‐Leucine Supplementation but Not Ketone Salts‐Taurine‐Leucine, Improves Endurance Cycling Performance,” International Journal of Sport Nutrition and Exercise Metabolism 32 (2022): 238–245. 35213817 10.1123/ijsnem.2021-0309

[34] A. J. Brady and B. Egan , “Acute Ingestion of a Ketone Monoester Without Co‐Ingestion of Carbohydrate Improves Running Economy in Male Endurance Runners,” Medicine and Science in Sports and Exercise 56 (2023): 134–142. 37565450 10.1249/MSS.0000000000003278

[35] H. S. Waldman , E. K. O'Neal , G. A. Barker , et al., “A Ketone Monoester With Carbohydrate Improves Cognitive Measures Postexercise, but Not Performance in Trained Females,” Medicine and Science in Sports and Exercise 56 (2024): 725–736. 38051034 10.1249/MSS.0000000000003352

[36] M. Gonzalez , C. Jachino , B. Murphy , et al., “The Effect of Acute Ketone Supplementation on Time to Fatigue in NCAA Division I Cross‐Country Athletes,” Nutraceuticals 4 (2024): 232–240.

[37] A. J. Brady , M. B. Moynagh , S. Devenney , and B. Egan , “Advanced Footwear Technology, but Not Acute Ingestion of a Ketone Monoester, Improves Running Economy in Middle‐ and Long‐Distance Runners,” Medicine and Science in Sports and Exercise 57 (2025): 1559–1569. 39999362 10.1249/MSS.0000000000003682

[38] C. D. Crabtree , J. Stoner , A. Buga , et al., “Cardiopulmonary Responses to Acute Exogenous Ketosis at Rest, and During Submaximal and Maximal Exercise,” Physiological Reports 13 (2025): e70397. 40443044 10.14814/phy2.70397 PMC12122769

[39] O'Malley T , E. Myette‐Cote , C. Durrer , and J. P. Little , “Nutritional Ketone Salts Increase Fat Oxidation but Impair High‐Intensity Exercise Performance in Healthy Adult Males,” Applied Physiology, Nutrition, and Metabolism 42 (2017): 1031–1035. 10.1139/apnm-2016-0641 28750585

[40] J. J. Leckey , M. L. Ross , M. Quod , J. A. Hawley , and L. M. Burke , “Ketone Diester Ingestion Impairs Time‐Trial Performance in Professional Cyclists,” Frontiers in Physiology 8 (2017): 806. 29109686 10.3389/fphys.2017.00806 PMC5660098

[41] C. Poffé , F. Wyns , M. Ramaekers , and P. Hespel , “Exogenous Ketosis Impairs 30‐Min Time‐Trial Performance Independent of Bicarbonate Supplementation,” Medicine and Science in Sports and Exercise 53 (2021): 1068–1078. 33196605 10.1249/MSS.0000000000002552 PMC8048725

[42] D. G. McCarthy , J. Bone , M. Fong , et al., “Acute Ketone Monoester Supplementation Impairs 20‐Min Time‐Trial Performance in Trained Cyclists: A Randomized, Crossover Trial,” International Journal of Sport Nutrition and Exercise Metabolism 33 (2023): 181–188. 37185454 10.1123/ijsnem.2022-0255

[43] E. E. Howard , J. T. Allen , J. L. Coleman , et al., “Ketone Monoester Plus Carbohydrate Supplementation Does Not Alter Exogenous and Plasma Glucose Oxidation or Metabolic Clearance Rate During Exercise in Men Compared With Carbohydrate Alone,” Journal of Nutrition 153 (2023): 1696–1709. 36893935 10.1016/j.tjnut.2023.03.002

[44] D. G. McCarthy , W. Bostad , J. Bone , F. J. Powley , D. L. Richards , and M. J. Gibala , “Effect of Acute Ketone Monoester Ingestion on Cardiorespiratory Responses to Exercise and the Influence of Blood Acidosis,” Medicine and Science in Sports and Exercise 55 (2023): 1286–1295. 36849121 10.1249/MSS.0000000000003141

[45] J. Bone , S. Baumgarten , D. G. McCarthy , W. Bostad , D. L. Richards , and M. J. Gibala , “Acute Ketone Monoester Supplementation Does Not Change Exercise Efficiency During Incremental Cycling in Trained Individuals,” Medicine and Science in Sports and Exercise 57 (2024): 163–172. 39186729 10.1249/MSS.0000000000003532

[46] M. L. Steinhauser , B. A. Olenchock , J. O'Keefe , et al., “The Circulating Metabolome of Human Starvation,” JCI Insight 3 (2018): e121434. 30135314 10.1172/jci.insight.121434 PMC6141167

[47] K. J. Kolnes , E. T. F. Nilsen , S. Brufladt , et al., “Effects of Seven Days' Fasting on Physical Performance and Metabolic Adaptation During Exercise in Humans,” Nature Communications 16 (2025): 122. 10.1038/s41467-024-55418-0 PMC11695724 39747857

[48] M. L. Kackley , J. A. Short , P. N. Hyde , et al., “A Pre‐Workout Supplement of Ketone Salts, Caffeine, and Amino Acids Improves High‐Intensity Exercise Performance in Keto‐Naïve and Keto‐Adapted Individuals,” Journal of the American College of Nutrition 39 (2020): 290–300. 32330107 10.1080/07315724.2020.1752846

[49] H. White , A. J. Heffernan , S. Worrall , A. Grunsfeld , and M. Thomas , “A Systematic Review of Intravenous β‐Hydroxybutyrate Use in Humans – A Promising Future Therapy?,” Frontiers in Medicine 8 (2021): 740374. 34621766 10.3389/fmed.2021.740374 PMC8490680

[50] P. J. Crowe , G. T. Royle , D. Wagner , and J. F. Burke , “Does Hyperketonemia Affect Protein or Glucose Kinetics in Postabsorptive or Traumatized Man?,” Journal of Surgical Research 47 (1989): 313–318. 2671503 10.1016/0022-4804(89)90141-8

[51] M. Beylot , D. Chassard , C. Chambrier , et al., “Metabolic Effects of a D‐Beta‐Hydroxybutyrate Infusion in Septic Patients: Inhibition of Lipolysis and Glucose Production but Not Leucine Oxidation,” Critical Care Medicine 22 (1994): 1091–1098. 8026196 10.1097/00003246-199407000-00007

[52] K. H. Mikkelsen , T. Seifert , N. H. Secher , T. Grondal , and G. van Hall , “Systemic, Cerebral and Skeletal Muscle Ketone Body and Energy Metabolism During Acute Hyper‐D‐Beta‐Hydroxybutyratemia in Post‐Absorptive Healthy Males,” Journal of Clinical Endocrinology and Metabolism 100 (2015): 636–643. 25415176 10.1210/jc.2014-2608

[53] M. Svart , L. C. Gormsen , J. Hansen , et al., “Regional Cerebral Effects of Ketone Body Infusion With 3‐Hydroxybutyrate in Humans: Reduced Glucose Uptake, Unchanged Oxygen Consumption and Increased Blood Flow by Positron Emission Tomography. A Randomized, Controlled Trial,” PLoS One 13 (2018): e0190556. 29489818 10.1371/journal.pone.0190556 PMC5830038

[54] A. Hiraide , M. Katayama , H. Sugimoto , T. Yoshioka , and T. Sugimoto , “Effect of 3‐Hydroxybutyrate on Posttraumatic Metabolism in Man,” Surgery 109 (1991): 176–181. 1992551

[55] A. K. Taggart , J. Kero , X. Gan , et al., “(D)‐Beta‐Hydroxybutyrate Inhibits Adipocyte Lipolysis via the Nicotinic Acid Receptor PUMA‐G,” Journal of Biological Chemistry 280 (2005): 26649–26652. 15929991 10.1074/jbc.C500213200

[56] R. S. Sherwin , R. G. Hendler , and P. Felig , “Effect of Ketone Infusions on Amino Acid and Nitrogen Metabolism in Man,” Journal of Clinical Investigation 55 (1975): 1382–1390. 1133179 10.1172/JCI108057 PMC301893

[57] K. S. Nair , S. L. Welle , D. Halliday , and R. G. Campbell , “Effect of Beta‐Hydroxybutyrate on Whole‐Body Leucine Kinetics and Fractional Mixed Skeletal Muscle Protein Synthesis in Humans,” Journal of Clinical Investigation 82 (1988): 198–205. 3392207 10.1172/JCI113570 PMC303494

[58] H. H. Thomsen , N. Rittig , M. Johannsen , et al., “Effects of 3‐Hydroxybutyrate and Free Fatty Acids on Muscle Protein Kinetics and Signaling During LPS‐Induced Inflammation in Humans: Anticatabolic Impact of Ketone Bodies,” American Journal of Clinical Nutrition 108 (2018): 857–867. 30239561 10.1093/ajcn/nqy170

[59] B. J. Stubbs , P. J. Cox , R. D. Evans , et al., “On the Metabolism of Exogenous Ketones in Humans,” Frontiers in Physiology 8 (2017): 848. 29163194 10.3389/fphys.2017.00848 PMC5670148

[60] T. Vandoorne , S. De Smet , M. Ramaekers , et al., “Intake of a Ketone Ester Drink During Recovery From Exercise Promotes mTORC1 Signaling but Not Glycogen Resynthesis in Human Muscle,” Frontiers in Physiology 8 (2017): 310. 28588499 10.3389/fphys.2017.00310 PMC5440563

[61] D. A. Holdsworth , P. J. Cox , T. Kirk , H. Stradling , S. G. Impey , and K. Clarke , “A Ketone Ester Drink Increases Postexercise Muscle Glycogen Synthesis in Humans,” Medicine and Science in Sports and Exercise 49 (2017): 1789–1795. 28398950 10.1249/MSS.0000000000001292 PMC5556006

[62] É. Myette‐Côté , H. Neudorf , H. Rafiei , K. Clarke , and J. P. Little , “Prior Ingestion of Exogenous Ketone Monoester Attenuates the Glycaemic Response to an Oral Glucose Tolerance Test in Healthy Young Individuals,” Journal of Physiology 596 (2018): 1385–1395. 29446830 10.1113/JP275709 PMC5899975

[63] B. J. Stubbs , P. J. Cox , R. D. Evans , M. Cyranka , K. Clarke , and H. de Wet , “A Ketone Ester Drink Lowers Human Ghrelin and Appetite,” Obesity (Silver Spring) 26 (2018): 269–273. 29105987 10.1002/oby.22051 PMC5813183

[64] C. Poffé , R. Robberechts , T. Podlogar , M. Kusters , T. Debevec , and P. Hespel , “Exogenous Ketosis Increases Blood and Muscle Oxygenation but Not Performance During Exercise in Hypoxia,” American Journal of Physiology. Regulatory, Integrative and Comparative Physiology 321 (2021): R844–R857. 34668436 10.1152/ajpregu.00198.2021

[65] M. Svart , N. Rittig , S. B. Pedersen , N. Jessen , and N. Møller , “Oral 3‐Hydroxybutyrate Ingestion Decreases Endogenous Glucose Production, Lipolysis, and Hormone‐Sensitive Lipase Phosphorylation in Adipose Tissue in Men: A Human Randomized, Controlled, Crossover Trial,” Diabetic Medicine 38 (2021): e14385. 32794582 10.1111/dme.14385

[66] R. Robberechts , G. Albouy , P. Hespel , and C. Poffè , “Exogenous Ketosis Improves Sleep Efficiency and Counteracts the Decline in REM Sleep Following Strenuous Exercise,” Medicine and Science in Sports and Exercise 55 (2023): 2064–2074. 37259248 10.1249/MSS.0000000000003231 PMC10581428

[67] E. M. Evans , J. P. Walhin , A. Hengist , J. A. Betts , D. J. Dearlove , and J. T. Gonzalez , “Ketone Monoester Ingestion Increases Postexercise Serum Erythropoietin Concentrations in Healthy Men,” American Journal of Physiology Endocrinology and Metabolism 324 (2023): E56–E61. 36449571 10.1152/ajpendo.00264.2022 PMC9870573

[68] M. Stalmans , D. Tominec , W. Lauriks , R. Robberechts , T. Debevec , and C. Poffé , “Exogenous Ketosis Attenuates Acute Mountain Sickness and Mitigates Normobaric High‐Altitude Hypoxemia,” Journal of Applied Physiology (1985) 137 (2024): 1301–1312. 10.1152/japplphysiol.00190.2024 39323395

[69] T. S. McClure , J. Phillips , A. P. Koutnik , et al., “Ketone Monoester Attenuates Declines in Cognitive Performance and Oxygen Saturation During Acute Severe Hypoxic Exposure Under Resting Conditions,” Experimental Physiology 109 (2024): 1672–1682. 39190580 10.1113/EP091794 PMC11442756

[70] S. J. Hannaian , J. Lov , S. E. Hawley , et al., “Acute Ingestion of a Ketone Monoester, Whey Protein, or Their Co‐Ingestion in the Overnight Postabsorptive State Elicit a Similar Stimulation of Myofibrillar Protein Synthesis Rates in Young Males: A Double‐Blind Randomized Trial,” American Journal of Clinical Nutrition 119 (2024): 716–729. 38215886 10.1016/j.ajcnut.2024.01.004 PMC10972741

[71] T. S. McClure , J. D. Buxton , B. Egan , et al., “Multisystem Impact of Altering Acid Load of Ingested Exogenous Ketone Supplements at Rest in Young Healthy Adults,” American Journal of Physiology. Regulatory, Integrative and Comparative Physiology 328 (2025): R386–r395. 40035490 10.1152/ajpregu.00057.2024

[72] I. Kimura , D. Inoue , T. Maeda , et al., “Short‐Chain Fatty Acids and Ketones Directly Regulate Sympathetic Nervous System via G Protein‐Coupled Receptor 41 (GPR41),” Proceedings of the National Academy of Sciences of the United States of America 108 (2011): 8030–8035. 21518883 10.1073/pnas.1016088108 PMC3093469

[73] J. Miyamoto , R. Ohue‐Kitano , H. Mukouyama , et al., “Ketone Body Receptor GPR43 Regulates Lipid Metabolism Under Ketogenic Conditions,” Proceedings of the National Academy of Sciences of the United States of America 116 (2019): 23813–23821. 31685604 10.1073/pnas.1912573116 PMC6876247

[74] T. Shimazu , M. D. Hirschey , J. Newman , et al., “Suppression of Oxidative Stress by Beta‐Hydroxybutyrate, an Endogenous Histone Deacetylase Inhibitor,” Science 339 (2013): 211–214. 23223453 10.1126/science.1227166 PMC3735349

[75] Z. Xie , D. Zhang , D. Chung , et al., “Metabolic Regulation of Gene Expression by Histone Lysine β‐Hydroxybutyrylation,” Molecular Cell 62 (2016): 194–206. 27105115 10.1016/j.molcel.2016.03.036 PMC5540445

[76] Y. H. Youm , K. Y. Nguyen , R. W. Grant , et al., “The Ketone Metabolite Beta‐Hydroxybutyrate Blocks NLRP3 Inflammasome‐Mediated Inflammatory Disease,” Nature Medicine 21 (2015): 263–269. 10.1038/nm.3804 PMC4352123 25686106

[77] J. Chen , Z. Li , Y. Zhang , et al., “Mechanism of Reduced Muscle Atrophy via Ketone Body (D)‐3‐Hydroxybutyrate,” Cell & Bioscience 12 (2022): 94. 35725651 10.1186/s13578-022-00826-2 PMC9208164

[78] X. Qiu , W. Wu , S. Zhang , C. Huang , and D. Lin , “3‐Hydroxybutyrate Promotes Myoblast Proliferation and Differentiation Through Energy Metabolism and GPR109a‐Mediated ca(2+)‐NFAT Signaling Pathways,” Journal of Proteome Research 24 (2025): 2063–2080. 40099866 10.1021/acs.jproteome.4c01150

[79] X. Zou , J. Meng , L. Li , et al., “Acetoacetate Accelerates Muscle Regeneration and Ameliorates Muscular Dystrophy in Mice,” Journal of Biological Chemistry 291 (2016): 2181–2195. 26645687 10.1074/jbc.M115.676510 PMC4732204

[80] B. A. Parker , C. M. Walton , S. T. Carr , et al., “β‐Hydroxybutyrate Elicits Favorable Mitochondrial Changes in Skeletal Muscle,” International Journal of Molecular Sciences 19 (2018): 2247. 30071599 10.3390/ijms19082247 PMC6121962

[81] H. Khouri , M. Roberge , J. R. Ussher , and C. Aguer , “Acetoacetate and d‐ and l‐β‐Hydroxybutyrate Have Distinct Effects on Basal and Insulin‐Stimulated Glucose Uptake in L6 Skeletal Muscle Cells,” American Journal of Physiology‐Cell Physiology 326 (2024): C1710–C1720. 38708524 10.1152/ajpcell.00718.2023

[82] B. Egan and A. P. Sharples , “Molecular Responses to Acute Exercise and Their Relevance for Adaptations in Skeletal Muscle to Exercise Training,” Physiological Reviews 103 (2023): 2057–2170. 36395350 10.1152/physrev.00054.2021

[83] L. M. Burke and J. A. Hawley , “Swifter, Higher, Stronger: What's on the Menu?,” Science 362 (2018): 781–787. 30442803 10.1126/science.aau2093

[84] E. Borsheim and R. Bahr , “Effect of Exercise Intensity, Duration and Mode on Post‐Exercise Oxygen Consumption,” Sports Medicine 33 (2003): 1037–1060. 14599232 10.2165/00007256-200333140-00002

[85] L. M. Burke , L. J. C. van Loon , and J. A. Hawley , “Postexercise Muscle Glycogen Resynthesis in Humans,” Journal of Applied Physiology (1985) 122 (2017): 1055–1067. 10.1152/japplphysiol.00860.2016 27789774

[86] A. M. Lundsgaard , A. M. Fritzen , and B. Kiens , “The Importance of Fatty Acids as Nutrients During Post‐Exercise Recovery,” Nutrients 12 (2020): 280. 31973165 10.3390/nu12020280 PMC7070550

[87] M. Beelen , L. M. Burke , M. J. Gibala , and L. J. van Loon , “Nutritional Strategies to Promote Postexercise Recovery,” International Journal of Sport Nutrition and Exercise Metabolism 20 (2010): 515–532. 21116024 10.1123/ijsnem.20.6.515

[88] G. H. Evans , L. J. James , S. M. Shirreffs , and R. J. Maughan , “Optimizing the Restoration and Maintenance of Fluid Balance After Exercise‐Induced Dehydration,” Journal of Applied Physiology (1985) 122 (2017): 945–951. 10.1152/japplphysiol.00745.2016 28126906

[89] H. H. K. Fullagar , G. E. Vincent , M. McCullough , S. Halson , and P. Fowler , “Sleep and Sport Performance,” Journal of Clinical Neurophysiology 40 (2023): 408–416. 36930212 10.1097/WNP.0000000000000638

[90] S. L. Halson , “Sleep in Elite Athletes and Nutritional Interventions to Enhance Sleep,” Sports Medicine 44, no. Suppl 1 (2014): S13–S23. 24791913 10.1007/s40279-014-0147-0 PMC4008810

[91] D. T. Thomas , K. A. Erdman , and L. M. Burke , “Position of the Academy of Nutrition and Dietetics, Dietitians of Canada, and the American College of Sports Medicine: Nutrition and Athletic Performance,” Medicine and Science in Sports and Exercise 48 (2016): 543–568. 26891166 10.1249/MSS.0000000000000852

[92] T. Stellingwerff , J. P. Morton , and L. M. Burke , “A Framework for Periodized Nutrition for Athletics,” International Journal of Sport Nutrition and Exercise Metabolism 29 (2019): 141–151. 30632439 10.1123/ijsnem.2018-0305

[93] S. B. Wilkinson , S. M. Phillips , P. J. Atherton , et al., “Differential Effects of Resistance and Endurance Exercise in the Fed State on Signalling Molecule Phosphorylation and Protein Synthesis in Human Muscle,” Journal of Physiology 586 (2008): 3701–3717. 18556367 10.1113/jphysiol.2008.153916 PMC2538832

[94] C. E. Donges , N. A. Burd , R. Duffield , et al., “Concurrent Resistance and Aerobic Exercise Stimulates Both Myofibrillar and Mitochondrial Protein Synthesis in Sedentary Middle‐Aged Men,” Journal of Applied Physiology (1985) 112, no. 12 (2012): 1992–2001. 10.1152/japplphysiol.00166.2012 22492939

[95] N. A. Burd , R. J. Andrews , D. W. West , et al., “Muscle Time Under Tension During Resistance Exercise Stimulates Differential Muscle Protein Sub‐Fractional Synthetic Responses in Men,” Journal of Physiology 590 (2012): 351–362. 22106173 10.1113/jphysiol.2011.221200 PMC3285070

[96] B. F. Miller and K. L. Hamilton , “A Perspective on the Determination of Mitochondrial Biogenesis,” American Journal of Physiology. Endocrinology and Metabolism 302 (2012): E496–E499. 22205627 10.1152/ajpendo.00578.2011 PMC3311289

[97] C. McGlory , S. van Vliet , T. Stokes , B. Mittendorfer , and S. M. Phillips , “The Impact of Exercise and Nutrition on the Regulation of Skeletal Muscle Mass,” Journal of Physiology 597 (2019): 1251–1258. 30010196 10.1113/JP275443 PMC6395419

[98] V. C. Figueiredo , “Revisiting the Roles of Protein Synthesis During Skeletal Muscle Hypertrophy Induced by Exercise,” American Journal of Physiology. Regulatory, Integrative and Comparative Physiology 317 (2019): R709–r718. 31508978 10.1152/ajpregu.00162.2019

[99] F. W. Booth and D. B. Thomason , “Molecular and Cellular Adaptation of Muscle in Response to Exercise: Perspectives of Various Models,” Physiological Reviews 71 (1991): 541–585. 2006222 10.1152/physrev.1991.71.2.541

[100] M. J. MacInnis and M. J. Gibala , “Physiological Adaptations to Interval Training and the Role of Exercise Intensity,” Journal of Physiology 595 (2017): 2915–2930. 27748956 10.1113/JP273196 PMC5407969

[101] M. D. Roberts , J. J. McCarthy , T. A. Hornberger , et al., “Mechanisms of Mechanical Overload‐Induced Skeletal Muscle Hypertrophy: Current Understanding and Future Directions,” Physiological Reviews 103 (2023): 2679–2757. 37382939 10.1152/physrev.00039.2022 PMC10625844

[102] J. A. Hawley , C. Lundby , J. D. Cotter , and L. M. Burke , “Maximizing Cellular Adaptation to Endurance Exercise in Skeletal Muscle,” Cell Metabolism 27 (2018): 962–976. 29719234 10.1016/j.cmet.2018.04.014

[103] O. C. Witard , S. R. Jackman , L. Breen , K. Smith , A. Selby , and K. D. Tipton , “Myofibrillar Muscle Protein Synthesis Rates Subsequent to a Meal in Response to Increasing Doses of Whey Protein at Rest and After Resistance Exercise,” American Journal of Clinical Nutrition 99 (2014): 86–95. 24257722 10.3945/ajcn.112.055517

[104] T. A. Churchward‐Venne , P. J. M. Pinckaers , J. S. J. Smeets , et al., “Dose‐Response Effects of Dietary Protein on Muscle Protein Synthesis During Recovery From Endurance Exercise in Young Men: A Double‐Blind Randomized Trial,” American Journal of Clinical Nutrition 112 (2020): 303–317. 32359142 10.1093/ajcn/nqaa073 PMC7398777

[105] J. E. Mallinson , S. L. Wardle , T. J. O'Leary , et al., “Protein Dose Requirements to Maximize Skeletal Muscle Protein Synthesis After Repeated Bouts of Resistance Exercise in Young Trained Women,” Scandinavian Journal of Medicine & Science in Sports 33 (2023): 2470–2481. 37787091 10.1111/sms.14506

[106] J. Trommelen , G. A. A. van Lieshout , J. Nyakayiru , et al., “The Anabolic Response to Protein Ingestion During Recovery From Exercise Has no Upper Limit in Magnitude and Duration In Vivo in Humans,” Cell Reports Medicine 4 (2023): 101324. 38118410 10.1016/j.xcrm.2023.101324 PMC10772463

[107] B. Egan , “Protein Intake for Athletes and Active Adults: Current Concepts and Controversies,” Nutrition Bulletin 41 (2016): 202–213.

[108] O. C. Witard , M. Hearris , and P. T. Morgan , “Protein Nutrition for Endurance Athletes: A Metabolic Focus on Promoting Recovery and Training Adaptation,” Sports Medicine 55 (2025): 1361–1376. 40117058 10.1007/s40279-025-02203-8 PMC12152099

[109] L. J. van Loon , W. H. Saris , M. Kruijshoop , and A. J. Wagenmakers , “Maximizing Postexercise Muscle Glycogen Synthesis: Carbohydrate Supplementation and the Application of Amino Acid or Protein Hydrolysate Mixtures,” American Journal of Clinical Nutrition 72 (2000): 106–111. 10871568 10.1093/ajcn/72.1.106

[110] K. E. Cogan , M. Evans , E. Iuliano , et al., “Co‐Ingestion of Protein or a Protein Hydrolysate With Carbohydrate Enhances Anabolic Signaling, but Not Glycogen Resynthesis, Following Recovery From Prolonged Aerobic Exercise in Trained Cyclists,” European Journal of Applied Physiology 118 (2018): 349–359. 29214461 10.1007/s00421-017-3775-x

[111] E. Mosquera‐Lopez , J. Louis , J. P. Edwards , et al., “Acute Nutritional Ketosis During Early Recovery From Aerobic Exercise Does Not Affect Skeletal Muscle Transcriptomic Response in Humans,” European Journal of Applied Physiology (2025): 10.1007/s00421-025-05987-9. PMC12948831 40960642

[112] Y. Takahashi , S. Terada , M. Banjo , K. Seike , S. Nakano , and H. Hatta , “Effects of β‐Hydroxybutyrate Treatment on Glycogen Repletion and Its Related Signaling Cascades in Epitrochlearis Muscle During 120 Min of Postexercise Recovery,” Applied Physiology, Nutrition, and Metabolism 44 (2019): 1311–1319. 10.1139/apnm-2018-0860 31051088

[113] Y. Takahashi , T. Matsumoto , W. Wang , T. Inaba , S. Terada , and H. Hatta , “Postexercise Ketone Monoester Administration Concomitant With Glucose Stimulates Glycogen Repletion in Soleus Muscle in Mice,” American Journal of Physiology. Cell Physiology 329 (2025): C366–C376. 40560777 10.1152/ajpcell.00311.2025

[114] E. Z. Maizels , N. B. Ruderman , M. N. Goodman , and D. Lau , “Effect of Acetoacetate on Glucose Metabolism in the Soleus and Extensor Digitorum Longus Muscles of the Rat,” Biochemical Journal 162 (1977): 557–568. 869905 10.1042/bj1620557 PMC1164638

[115] M. D. Quinones , K. Weiman , and P. W. R. Lemon , “Ketone Monoester Followed by Carbohydrate Ingestion After Glycogen‐Lowering Exercise Does Not Improve Subsequent Endurance Cycle Time Trial Performance,” Nutrients 16 (2024): 932. 38612966 10.3390/nu16070932 PMC11013615

[116] L. Clarke , S. L. Russell , B. Spellanzon , J. L. Maher , J. A. Betts , and J. T. Gonzalez , “Ketone Ester Ingestion Increases Exogenous Carbohydrate Storage and Lowers Glycemia During Post‐Exercise Recovery: A Randomised Crossover Trial,” European Journal of Nutrition 64 (2025): 253. 40794305 10.1007/s00394-025-03784-w PMC12343750

[117] J. R. Thompson and G. Wu , “The Effect of Ketone Bodies on Nitrogen Metabolism in Skeletal Muscle,” Comparative Biochemistry and Physiology. B 100 (1991): 209–216. 10.1016/0305-0491(91)90363-i 1799962

[118] S. J. Hannaian , J. Lov , Z. Cheng‐Boivin , et al., “Acute Effects of a Ketone Monoester, Whey Protein, or Their Coingestion on mTOR Trafficking and Protein‐Protein Colocalization in Human Skeletal Muscle,” American Journal of Physiology. Cell Physiology 326 (2024): C1769–c1775. 38682238 10.1152/ajpcell.00207.2024 PMC11371313

[119] M. Mose , K. Brodersen , N. Rittig , et al., “Anabolic Effects of Oral Leucine‐Rich Protein With and Without β‐Hydroxybutyrate on Muscle Protein Metabolism in a Novel Clinical Model of Systemic Inflammation – A Randomized Crossover Trial,” American Journal of Clinical Nutrition 114 (2021): 1159–1172. 34081111 10.1093/ajcn/nqab148

[120] C. Hiroux , M. Schouten , I. de Glisezinski , et al., “Effect of Increased Protein Intake and Exogenous Ketosis on Body Composition, Energy Expenditure and Exercise Capacity During a Hypocaloric Diet in Recreational Female Athletes,” Frontiers in Physiology 13 (2022): 1063956. 36714318 10.3389/fphys.2022.1063956 PMC9880233

[121] C. Poffé , M. Ramaekers , R. Van Thienen , and P. Hespel , “Ketone Ester Supplementation Blunts Overreaching Symptoms During Endurance Training Overload,” Journal of Physiology 597 (2019): 3009–3027. 31039280 10.1113/JP277831 PMC6851819

[122] D. J. Dearlove , A. Soto Mota , D. Hauton , et al., “The Effects of Endogenously‐ and Exogenously‐Induced Hyperketonemia on Exercise Performance and Adaptation,” Physiological Reports 10 (2022): e15309. 35614576 10.14814/phy2.15309 PMC9133544

[123] G. Howatson and K. A. van Someren , “The Prevention and Treatment of Exercise‐Induced Muscle Damage,” Sports Medicine 38 (2008): 483–503. 18489195 10.2165/00007256-200838060-00004

[124] D. J. Owens , C. Twist , J. N. Cobley , G. Howatson , and G. L. Close , “Exercise‐Induced Muscle Damage: What Is It, What Causes It and What Are the Nutritional Solutions?,” European Journal of Sport Science 19 (2019): 71–85. 30110239 10.1080/17461391.2018.1505957

[125] D. R. Moore , S. M. Phillips , J. A. Babraj , K. Smith , and M. J. Rennie , “Myofibrillar and Collagen Protein Synthesis in Human Skeletal Muscle in Young Men After Maximal Shortening and Lengthening Contractions,” American Journal of Physiology. Endocrinology and Metabolism 288 (2005): E1153–E1159. 15572656 10.1152/ajpendo.00387.2004

[126] G. F. Pavis , T. S. O. Jameson , M. L. Dirks , et al., “Improved Recovery From Skeletal Muscle Damage Is Largely Unexplained by Myofibrillar Protein Synthesis or Inflammatory and Regenerative Gene Expression Pathways,” American Journal of Physiology. Endocrinology and Metabolism 320 (2021): E291–E305. 33284089 10.1152/ajpendo.00454.2020 PMC8260377

[127] S. M. Pasiakos , H. R. Lieberman , and T. M. McLellan , “Effects of Protein Supplements on Muscle Damage, Soreness and Recovery of Muscle Function and Physical Performance: A Systematic Review,” Sports Medicine 44 (2014): 655–670. 24435468 10.1007/s40279-013-0137-7

[128] H. R. Bae , D. H. Kim , M. H. Park , et al., “β‐Hydroxybutyrate Suppresses Inflammasome Formation by Ameliorating Endoplasmic Reticulum Stress via AMPK Activation,” Oncotarget 7 (2016): 66444–66454. 27661104 10.18632/oncotarget.12119 PMC5341812

[129] D. I. Benjamin , P. Both , J. S. Benjamin , et al., “Fasting Induces a Highly Resilient Deep Quiescent State in Muscle Stem Cells via Ketone Body Signaling,” Cell Metabolism 34 (2022): 902–918.e6. 35584694 10.1016/j.cmet.2022.04.012 PMC9177797

[130] P. W. Martin‐Arrowsmith , J. Lov , J. Dai , J. A. Morais , and T. A. Churchward‐Venne , “Ketone Monoester Supplementation Does Not Expedite the Recovery of Indices of Muscle Damage After Eccentric Exercise,” Frontiers in Nutrition 7 (2020): 607299. 33364251 10.3389/fnut.2020.607299 PMC7752861

[131] T. S. O. Jameson , H. Islam , B. T. Wall , J. P. Little , and F. B. Stephens , “Oral Ketone Monoester Supplementation Does Not Accelerate Recovery of Muscle Force or Modulate Circulating Cytokine Concentrations After Muscle‐Damaging Eccentric Exercise in Healthy Males and Females,” Experimental Physiology 107 (2022): 1339–1348. 36114653 10.1113/EP090546 PMC9828245

[132] C. Poffé , R. Robberechts , M. Stalmans , J. Vanderroost , S. Bogaerts , and P. Hespel , “Exogenous Ketosis Increases Circulating Dopamine Concentration and Maintains Mental Alertness in Ultra‐Endurance Exercise,” Journal of Applied Physiology (1985) 134 (2023): 1456–1469. 10.1152/japplphysiol.00791.2022 37141424

[133] T. R. Lundberg and G. Howatson , “Analgesic and Anti‐Inflammatory Drugs in Sports: Implications for Exercise Performance and Training Adaptations,” Scandinavian Journal of Medicine & Science in Sports 28 (2018): 2252–2262. 30102811 10.1111/sms.13275

[134] H. H. Fullagar , S. Skorski , R. Duffield , R. Julian , J. Bartlett , and T. Meyer , “Impaired Sleep and Recovery After Night Matches in Elite Football Players,” Journal of Sports Sciences 34 (2016): 1333–1339. 26750446 10.1080/02640414.2015.1135249

[135] D. E. Rae , T. Chin , K. Dikgomo , et al., “One Night of Partial Sleep Deprivation Impairs Recovery From a Single Exercise Training Session,” European Journal of Applied Physiology 117 (2017): 699–712. 28247026 10.1007/s00421-017-3565-5

[136] J. A. Vitale , G. Banfi , A. Galbiati , L. Ferini‐Strambi , and A. La Torre , “Effect of a Night Game on Actigraphy‐Based Sleep Quality and Perceived Recovery in Top‐Level Volleyball Athletes,” International Journal of Sports Physiology and Performance 14 (2019): 265–269. 30040006 10.1123/ijspp.2018-0194

[137] J. Leota , D. M. Presby , F. Le , et al., “Dose‐Response Relationship Between Evening Exercise and Sleep,” Nature Communications 16 (2025): 3297. 10.1038/s41467-025-58271-x PMC12000559 40234380

[138] J. Stutz , R. Eiholzer , and C. M. Spengler , “Effects of Evening Exercise on Sleep in Healthy Participants: A Systematic Review and Meta‐Analysis,” Sports Medicine 49 (2019): 269–287. 30374942 10.1007/s40279-018-1015-0

[139] I. Mujika , A. P. Sharma , and T. Stellingwerff , “Contemporary Periodization of Altitude Training for Elite Endurance Athletes: A Narrative Review,” Sports Medicine 49 (2019): 1651–1669. 31452130 10.1007/s40279-019-01165-y

[140] M. Stalmans , D. Tominec , R. Robberechts , et al., “A Single Night in Hypoxia Either With or Without Ketone Ester Ingestion Reduces Sleep Quality Without Impacting Next‐Day Exercise Performance,” Medicine and Science in Sports and Exercise 57 (2025): 807–819. 39809236 10.1249/MSS.0000000000003604 PMC11878631

[141] S. Chikahisa , N. Shimizu , T. Shiuchi , and H. Séi , “Ketone Body Metabolism and Sleep Homeostasis in Mice,” Neuropharmacology 79 (2014): 399–404. 24361452 10.1016/j.neuropharm.2013.12.009

[142] S. Katsuya , Y. Kawata , Y. Kawamura , J. Kawamura , and J. Tsubota , “Effect of D‐β‐Hydroxybutyrate on Sleep Quality in Healthy Participants: A Randomized, Double‐Blind, Placebo‐Controlled Study,” Bioscience, Biotechnology, and Biochemistry 89 (2025): 769–775. 39914452 10.1093/bbb/zbaf017

[143] D. Masi , M. E. Spoltore , R. Rossetti , et al., “The Influence of Ketone Bodies on Circadian Processes Regarding Appetite, Sleep and Hormone Release: A Systematic Review of the Literature,” Nutrients 14 (2022): 1410. 35406023 10.3390/nu14071410 PMC9002750

[144] S. A. Masino , T. Li , P. Theofilas , et al., “A Ketogenic Diet Suppresses Seizures in Mice Through Adenosine A_1_ Receptors,” Journal of Clinical Investigation 121 (2011): 2679–2683. 21701065 10.1172/JCI57813 PMC3223846

[145] Y. Daikhin and M. Yudkoff , “Ketone Bodies and Brain Glutamate and GABA Metabolism,” Developmental Neuroscience 20 (1998): 358–364. 9778572 10.1159/000017331

[146] A. Hone‐Blanchet , B. Antal , L. McMahon , et al., “Acute Administration of Ketone Beta‐Hydroxybutyrate Downregulates 7T Proton Magnetic Resonance Spectroscopy‐Derived Levels of Anterior and Posterior Cingulate GABA and Glutamate in Healthy Adults,” Neuropsychopharmacology 48 (2023): 797–805. 35995971 10.1038/s41386-022-01364-8 PMC10066400

[147] R. E. Brown , R. Basheer , J. T. McKenna , R. E. Strecker , and R. W. McCarley , “Control of Sleep and Wakefulness,” Physiological Reviews 92 (2012): 1087–1187. 22811426 10.1152/physrev.00032.2011 PMC3621793

[148] R. J. Maughan and J. B. Leiper , “Sodium Intake and Post‐Exercise Rehydration in Man,” European Journal of Applied Physiology and Occupational Physiology 71 (1995): 311–319. 8549573 10.1007/BF00240410

[149] S. M. Shirreffs and R. J. Maughan , “Volume Repletion After Exercise‐Induced Volume Depletion in Humans: Replacement of Water and Sodium Losses,” American Journal of Physiology 274 (1998): F868–F875. 9612323 10.1152/ajprenal.1998.274.5.F868

[150] R. Robberechts , C. Poffé , and P. Hespel , “Exogenous Ketosis Suppresses Diuresis and Atrial Natriuretic Peptide During Exercise,” Journal of Applied Physiology (1985) 133 (2022): 449–460. 10.1152/japplphysiol.00061.2022 35771216

[151] E. T. Vestergaard , N. B. Zubanovic , N. Rittig , et al., “Acute Ketosis Inhibits Appetite and Decreases Plasma Concentrations of Acyl Ghrelin in Healthy Young Men,” Diabetes, Obesity & Metabolism 23 (2021): 1834–1842. 10.1111/dom.14402 33852195

[152] K. M. Lauritsen , E. Søndergaard , M. Svart , N. Møller , and L. C. Gormsen , “Ketone Body Infusion Increases Circulating Erythropoietin and Bone Marrow Glucose Uptake,” Diabetes Care 41 (2018): e152–e154. 30327354 10.2337/dc18-1421

[153] C. Poffé , R. Robberechts , R. Van Thienen , and P. Hespel , “Exogenous Ketosis Elevates Circulating Erythropoietin and Stimulates Muscular Angiogenesis During Endurance Training Overload,” Journal of Physiology 601 (2023): 2345–2358. 37062892 10.1113/JP284346

[154] J. M. Kanta , A. Lundsgaard , A. Schaufuss , M. Kleinert , B. Kiens , and A. M. Fritzen , “Induction of Erythropoietin by Dietary Medium‐Chain Triacylglycerol in Humans,” American Journal of Physiology. Endocrinology and Metabolism 328 (2025): E210–e216. 39792092 10.1152/ajpendo.00415.2024

[155] E. E. Howard , J. T. Allen , J. L. McNiff , S. D. Small , K. S. O'Fallon , and L. M. Margolis , “Ketone Monoester Plus High‐Dose Glucose Supplementation Before Exercise Does Not Affect Immediate Post‐Exercise Erythropoietin Concentrations Versus Glucose Alone,” Physiological Reports 12 (2024): e70009. 39174870 10.14814/phy2.70009 PMC11341272

[156] C. Lundby and N. V. Olsen , “Effects of Recombinant Human Erythropoietin in Normal Humans,” Journal of Physiology 589 (2011): 1265–1271. 20807784 10.1113/jphysiol.2010.195917 PMC3082090

[157] R. A. Jacobs , P. Rasmussen , C. Siebenmann , et al., “Determinants of Time Trial Performance and Maximal Incremental Exercise in Highly Trained Endurance Athletes,” Journal of Applied Physiology (1985) 111 (2011): 1422–1430. 10.1152/japplphysiol.00625.2011 21885805

[158] W. F. J. Schmidt , T. Hoffmeister , N. Wachsmuth , and W. C. Byrnes , “Cobalt Misuse in Sports,” German Journal of Sports Medicine 70 (2019): 129–134.

[159] S. Elliott , E. Pham , and I. C. Macdougall , “Erythropoietins: A Common Mechanism of Action,” Experimental Hematology 36 (2008): 1573–1584. 18922615 10.1016/j.exphem.2008.08.003

[160] A. A. Robertson , B. M. Lynagh , K. M. A. Thompson , and D. G. McCarthy , “EPOtential Target for Endurance Performance: The Effect of Exogenous Ketone Supplementation on Circulating Erythropoietin Levels,” Journal of Physiology 601 (2023): 3991–3992. 37566813 10.1113/JP285257

[161] S. Lamon and A. P. Russell , “The Role and Regulation of Erythropoietin (EPO) and Its Receptor in Skeletal Muscle: How Much Do We Really Know?,” Frontiers in Physiology 4 (2013): 176. 23874302 10.3389/fphys.2013.00176 PMC3710958

[162] T. Thewlis , What's the Big Deal About Ketones?, accessed April 1, 2025, https://www.cyclingweekly.com/fitness/whats‐the‐big‐deal‐about‐ketones.

[163] M. Ghyselinck , “Was het toverdrank uit een wonderflesje dat Remco Evenepoel in één teug naar binnen goot? Wij zochten uit of je nu harder gaat fietsen van ketonen Het Laatste Nieuws,” accessed May 10, 2023, https://www.hln.be/wielrennen/was‐het‐toverdrank‐uit‐een‐wonderflesje‐dat‐remco‐evenepoel‐in‐een‐teug‐naar‐binnen‐goot‐wij‐zochten‐uit‐of‐je‐nu‐harder‐gaat‐fietsen‐van‐ketonen~a0c08b54/.

[164] D. J. Dearlove , O. K. Harrison , L. Hodson , A. Jefferson , K. Clarke , and P. J. Cox , “The Effect of Blood Ketone Concentration and Exercise Intensity on Exogenous Ketone Oxidation Rates in Athletes,” Medicine and Science in Sports and Exercise 53 (2021): 505–516. 32868580 10.1249/MSS.0000000000002502 PMC7886359

[165] D. J. Dearlove , D. Holdsworth , T. Kirk , et al., “β‐Hydroxybutyrate Oxidation in Exercise Is Impaired by Low‐Carbohydrate and High‐Fat Availability,” Frontiers in Medicine 8 (2021): 721673. 34901052 10.3389/fmed.2021.721673 PMC8655871

[166] R. Nielsen , N. Møller , L. C. Gormsen , et al., “Cardiovascular Effects of Treatment With the Ketone Body 3‐Hydroxybutyrate in Chronic Heart Failure Patients,” Circulation 139 (2019): 2129–2141. 30884964 10.1161/CIRCULATIONAHA.118.036459 PMC6493702

[167] A. J. Rourke , C. M. S. Yong , G. B. Coombs , et al., “Acute Ketone Monoester Ingestion Lowers Resting Cerebral Blood Flow: A Randomized Cross‐Over Trial,” Journal of Physiology (2025): 10.1113/JP287320. PMC12997017 40349325

